# Integrating PROSPECT-D physics and adversarial domain adaptation resnet for robust cross-ecosystem plant traits estimation

**DOI:** 10.3389/fpls.2025.1612430

**Published:** 2025-07-25

**Authors:** Hui Zhang, Haoxuan Su, Tie Shen, Guangyao Sun, Qi Wang

**Affiliations:** ^1^ School of Information, Guizhou University of Finance and Economics, Guiyang, China; ^2^ Guizhou Provincial Leading Talent Workstation for Protein Design and Biological Imaging Innovation, Key Laboratory of National Forestry and Grassland Administration on Biodiversity Conservation in Karst Mountainous Areas of Southwestern China, College of Life Science, Guizhou Normal University, Guiyang, China; ^3^ College of Information and Electrical Engineering, China Agricultural University, Beijing, China; ^4^ State Key Laboratory of Public Big Data, College of Computer Science and Technology, Guizhou University, Guiyang, China

**Keywords:** hyperspectral, deep learning, plant phenotyping, adversarial domain adaptation, plant functional traits

## Abstract

Plant functional traits, including chlorophyll content (CHL), equivalent water thickness (EWT), and leaf mass per area (LMA), are critical indicators for assessing ecosystem functioning, functional diversity, and their roles in the Earth system. Hyperspectral remote sensing serves as a pivotal tool for multi-trait mapping; however, existing methods exhibit limited generalizability across ecosystems, land cover types, and sensor modalities. Challenges such as data heterogeneity, domain shifts, and sparse *in situ* measurements further hinder model generalization. To address these limitations, this study developed PPADA-Net, a novel framework integrating PROSPECT-D radiative transfer modeling with adversarial domain adaptation for robust cross-ecosystem plant trait prediction. In a two-stage process, a residual network is pretrained on synthetic spectra from PROSPECT-D to capture biophysical links between leaf traits and spectral signatures, followed by adversarial learning to align source and target domain features, reducing domain shifts. The model’s performance is validated on four public datasets and one field-measured dataset. PPADA-Net outperforms traditional partial least squares regression (PLSR) and purely data-driven models (e.g., ResNet), achieving mean R² values of 0.72 (CHL),0.77 (EWT), and 0.86 (LMA). Additionally, PPADA-Net demonstrates practical utility in a real-world farmland dataset (D5), achieving high-precision spatial mapping with an nRMSE of 0.07 for LMA. By merging physical priors with adaptive learning, PPADA-Net enhances spectral-trait modeling under data scarcity, offering a scalable tool for ecosystem monitoring, precision agriculture, and climate adaptation.

## 1 Introduction

Global climate change and the sustainable management of ecosystems are major concerns for the international community. The 2015 Paris Agreement set a clear goal of limiting the global temperature increase to below 1.5°C, identifying carbon neutrality as a crucial strategy to achieve this objective. In this context, accurately monitoring vegetation health and functional dynamics is critically important, as vegetation serves as a primary carbon sink in terrestrial ecosystems (Fatichi et al., 2019). Thus, there is an urgent need for efficient, reliable methods to quantify plant functional traits, supporting carbon sink assessments, vegetation restoration monitoring, and precision agriculture.

Plant functional traits are critical indicators for understanding ecosystem dynamics, vegetation health, and biogeochemical cycles (Wright et al., 2004). For example, chlorophyll content (CHL), which quantifies leaf chlorophyll pigment concentration, directly reflects photosynthetic capacity and nitrogen status, making it vital for assessing plant productivity and stress responses (Meloni et al., 2003). Equivalent water thickness (EWT), defined as the mass of water per unit leaf area, offers insights into plant water-use efficiency and drought resilience, thereby informing irrigation management and climate adaptation strategies (Hunt and Rock, 1989). Leaf mass per area (LMA), representing the ratio of leaf dry mass to area, correlates with leaf longevity, carbon allocation, and environmental stress resistance, serving as a key parameter for modeling carbon sequestration and ecosystem functions (Poorter et al., 2009). Collectively, these traits underpin efforts to monitor global vegetation changes, predict agricultural yields, and mitigate climate impacts (Drenovsky et al., 2012). However, traditional measurement methods such as destructive sampling and laboratory analysis are labor-intensive, time-consuming, and low throughput, limiting their applicability across large spatial and temporal scales (Zhang et al., 2025b). Consequently, there is an urgent need for non-destructive, high-efficiency approaches to estimate plant traits rapidly and accurately, enabling real-time decision-making in precision agriculture, ecological conservation, and climate resilience initiatives.

Hyperspectral remote sensing has emerged as a powerful tool for non-destructive and high-throughput estimation of plant traits (Angel and Shiklomanov, 2022), providing rich spectral information across hundreds of narrow bands to detect subtle biochemical and physiological variations (Sun et al., 2025). For instance, Hoeppner et al. (2020) leveraged hyperspectral data to predict CHL in forest ecosystems by analyzing reflectance features in the visible-red edge regions (680–780 nm), achieving robust correlations with ground-truth measurements. Similarly, Shu et al. (2022) demonstrated the utility of hyperspectral images in estimating EWT for crops, enabling real-time drought monitoring in precision agriculture. These studies underscore hyperspectral imaging’s capability to resolve trait-specific spectral signatures. However, existing approaches primarily focus on single ecosystem applications (forests or croplands), where models are trained and validated in homogeneous environments. This limits their generalizability to heterogeneous ecosystems, such as transitions from controlled agricultural fields to natural grasslands or mixed forests, where spectral-trait relationships may vary significantly due to differences in species composition, canopy structure, and environmental stressors (Heidenreich and Richardson, 2020). Consequently, improving model transferability across ecosystems remains a critical challenge, necessitating frameworks that address spectral heterogeneity and domain shifts inherent to multi-environment applications.

Radiative transfer models (RTMs), such as the widely adopted PROSPECT model, offer a mechanistic framework for simulating vegetation reflectance spectra based on biophysical and biochemical properties (Haboudane et al., 2004). PROSPECT has been extensively used to retrieve key plant traits, including CHL, EWT, and LMA, by inversely linking observed hyperspectral data to simulated canopy reflectance (Jacquemoud and Baret, 1990). For example, Wang et al. (2015) demonstrated PROSPECT’s capability to estimate CHL, LMA and nitrogen content in leaves, leveraging its parameterization of leaf biochemistry and canopy architecture. This model enables researchers to disentangle complex interactions between light and vegetation, such as the influence of leaf dry matter on SWIR reflectance or water content on NIR absorption, thereby offering interpretable insights into spectral-trait relationships (Broge and Leblanc, 2001). However, PROSPECT’s practical application faces inherent limitations, most notably the ill-posed inverse problem: multiple combinations of input parameters can generate nearly identical canopy reflectance spectra (Jing et al., 2004), leading to non-unique solutions and heightened uncertainty in trait retrieval. Additionally, the model’s performance depends critically on accurate prior knowledge of species-specific parameters, which may vary significantly across ecosystems or under stress conditions. These challenges underscore the need for hybrid approaches that integrate physical models with data-driven techniques to enhance robustness and scalability in trait estimation.

Data-driven approaches, particularly machine learning (ML) and deep learning (DL), have gained prominence in plant trait estimation by leveraging spectral data to establish empirical relationships between reflectance and biochemical properties (Sun et al., 2024; Zhang et al., 2021). Traditional ML methods, such as partial least squares regression (PLSR) and random forest (RF), have been successfully used to predict traits like LMA by integrating spectral reflectance and vegetation indices. For instance, Helsen et al. (2021) explored the potential of hyperspectral leaf reflectance-based PLSR model to predict LMA and EWT at the intraspecific level for two herbs and two shrubs. Similarly, Yin et al. (2023) employed RF to map CHL in crops by combining multispectral features with environmental covariates, demonstrating the flexibility of ML in handling high-dimensional data. However, these models often fail when applied to novel environments, where trait-spectra relationships differ substantially. Deep learning offers a promising alternative by automating hierarchical feature extraction and capturing nonlinear interactions. For example, Yue et al. (2024) developed a convolutional neural network (CNN) to estimate leaf chlorophyll content using hyperspectral reflectance data, while the CNN excels at prediction in each individual growth stage. Despite these advances, DL models face two critical challenges: (1) training robust networks requires extensive field measurements, which are labor-intensive to collect for traits like LMA or EWT, and (2) spectral features extracted from one ecosystem may misalign with those from another, degrading prediction accuracy in cross-domain scenarios. These limitations underscore the need for adaptive frameworks that integrate data-driven learning with domain-invariant representations to enable reliable trait estimation across heterogeneous environments.

Transfer learning is a paradigm that leverages knowledge from source domains to improve model performance in target domains with limited labeled data, offers a transformative solution to address domain shifts in spectral-trait modeling (Pan and Yang, 2010). Traditional trait estimation models, including physically based radiative transfer models (RTMs) such as PROSPECT, and empirical data-driven approaches like PLSR and RF, have shown effectiveness within homogeneous ecosystems but face significant challenges in cross-ecosystem applications. RTMs suffer from the ill-posed inverse problem and rely heavily on accurate species-specific parameterization, which is often unavailable or inaccurate across diverse environments, resulting in ambiguous and uncertain trait retrieval. Similarly, data-driven models tend to perform poorly when spectral-trait relationships vary due to differences in species composition, canopy structure, and environmental conditions, limiting their generalizability and practical applicability across heterogeneous landscapes. By reusing pre-trained features or aligning feature distributions across domains, transfer learning reduces dependency on large target-domain datasets while enhancing generalization (Radford et al., 2021). For example, adversarial domain adaptation, a subfield of transfer learning, employs domain-discriminative networks to reduce discrepancies between source and target feature spaces. Ganin et al. (2016) demonstrated this approach’s efficacy in computer vision through Domain-Adversarial Neural Networks (DANN), where adversarial training aligned image features across disparate datasets, achieving 20% higher accuracy in cross-domain tasks. In plant trait estimation, such techniques hold promise for bridging spectral heterogeneity across ecosystems. Bhadra et al. (2024) proposed a transfer learning approach combined with PROSAIL simulated data to achieve accurate prediction of CHL and average leaf angle based on UAV hyperspectral imagery. However, their application remains underexplored, particularly in scenarios where spectral signatures diverge due to variations in species composition, canopy structure, or environmental conditions. For example, while models trained on agricultural crop spectra may struggle to generalize to forest ecosystems, adversarial learning could theoretically align domain-invariant traits to mitigate such discrepancies (Amirkolaee et al., 2024).

Despite this potential, few studies have systematically evaluated transfer learning’s capacity to enhance cross-ecosystem trait prediction, leaving critical gaps in understanding how to optimize domain adaptation for vegetation monitoring, limiting their applicability across ecosystems with varying species composition, canopy structures, and environmental conditions. Existing data-driven models often function as black boxes, providing limited interpretability and performing poorly under data scarcity, which restricts their applicability across ecosystems with varying species compositions, canopy structures, and environmental conditions. In contrast, physical models such as PROSPECT-D are grounded in well-established biophysical principles but suffer from issues of non-uniqueness and sensitivity to prior assumptions, particularly in heterogeneous landscapes. These complementary strengths and limitations suggest a promising direction: integrating physical modeling with adversarial domain adaptation. Specifically, pretraining on synthetic spectra–trait pairs generated by PROSPECT-D introduces biophysical priors into the model, while adversarial learning facilitates the alignment of cross-domain representations, thereby enhancing generalization under spectral heterogeneity. In summary, the primary objectives of this study are: (1) to evaluate the effectiveness of integrating data-driven models and physical models for plant trait prediction using hyperspectral data; (2) to develop a transfer learning framework based on adversarial domain adaptation to improve model generalization and transferability across heterogeneous environmental conditions; (3) to validate the proposed models and methodologies on real-world crop datasets, assessing their practical applicability and robustness across diverse field conditions.

## 2 Materials and methods

### 2.1 Dataset collection

This study employs five independent datasets (**Table 1**) from distinct ecosystems, each containing measurements of CHL, EWT, LMA, and corresponding leaf reflectance spectra. All spectral data were acquired using a high-resolution spectroradiometer (1 nm resolution). The LMA, CHL, and EWT values in each dataset were determined following standardized protocols, with LMA calculated as the ratio of leaf dry mass to projected area. Dataset 1 (D1) comprises data from five forest plant species predominantly distributed in temperate regions. Dataset 2 (D2) contains six tropical plant species spanning subtropical and tropical ecosystems. Dataset 3 (D3) includes two herbaceous species cultivated under controlled laboratory conditions. Dataset 4 (D4) incorporates two xerophytic plant species adapted to arid environments with water-limited growth conditions. These four datasets (D1–D4) are publicly available and can be accessed through the EcoSIS (Ecological Spectral Information System) platform at https://ecosis.org.

**Table 1:** Statistical description of the dataset.

Dataset	Instrument	Spectral range	No. species	Number of samples
D1	ASD FieldSpec3	350-2500	5	212
D2	Perkin Elmer Lambda-19	400-2400	6	356
D3	SVC HR 1024i	350-2500	2	178
D4	ASD FieldSpec	400-2450	2	251
D5(ours)	ASD FieldSpec3	350-2500	3	490

**Figure 1:**
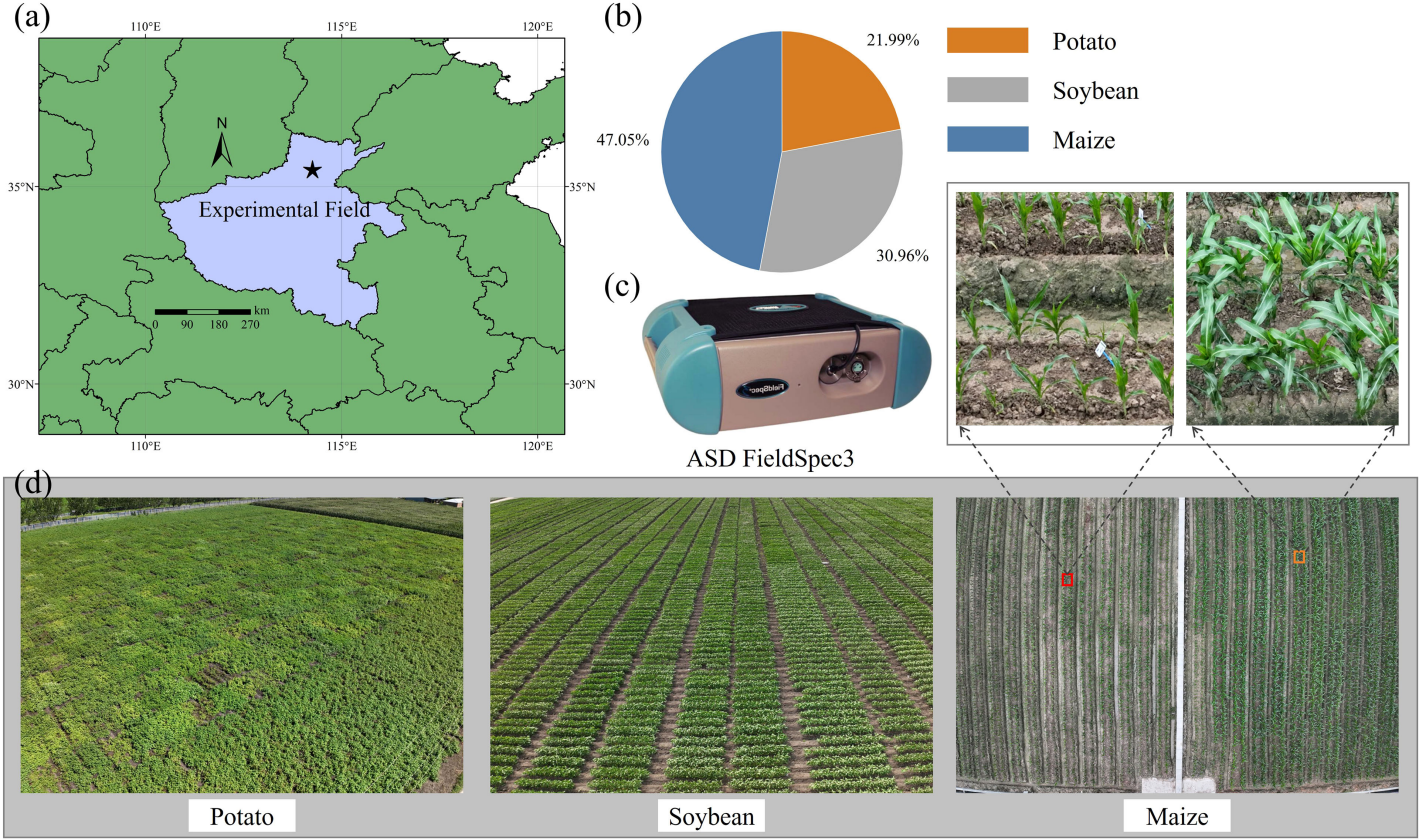
**(a)** Experiment location of this study, **(b)** proportion of three crop samples measured on site **(c)** spectral acquisition instrument, **(d)** field images of three crops.

Dataset 5 (D5) was collected from an agricultural research station in Xinxiang, Henan Province, China (113°45′40″E, 35°8′11″N) (**Figure 1a**), and comprises three crop species: potato, soybean, and maize. Among these, soybean and maize were part of breeding trials and were planted in separate plots, with each plot corresponding to a unique cultivar, including 36 cultivars for maize and 151 for soybean (**Figure 1b**). Notably, maize was sown in two separate batches, 28 days apart, resulting in distinct growth stages between the two groups of maize plots (**Figure 1d**). All crops were managed according to local agricultural practices, with optimal fertilization, pest control, and field maintenance applied. Spectral measurements were conducted on fresh leaves using standardized protocols to ensure data quality and comparability.

Spectral measurements were conducted using an ASD FieldSpec3 spectroradiometer (Analytical Spectral Devices Inc., Boulder, CO, USA) (**Figure 1c**), which operates over a spectral range of 350–2500 nm with a 1 nm sampling interval. Prior to data collection, the instrument was calibrated with a Spectralon white reference panel to ensure accurate reflectance measurements. All measurements were performed between 10:00 AM and 2:00 PM local time under clear-sky conditions to minimize the effects of solar angles and atmospheric variability. For each crop species, fully expanded, healthy leaves were selected from the upper canopy to maintain consistency and physiological relevance. The adaxial surface of each leaf was positioned perpendicular to the optical fiber probe, and a leaf clip equipped with an internal light source was used to provide stable illumination and reduce ambient light interference. For each leaf, three replicate spectral measurements were acquired and averaged to reduce noise and enhance signal reliability.

CHL was determined using a solvent extraction method with 95% ethanol, and pigment concentrations were quantified spectrophotometrically based on absorbance at 649 nm and 665 nm, following established protocols. Leaf fresh weight and area were measured using an electronic balance and a leaf area meter, respectively. Samples were then oven-dried at 65°C for 48 hours to determine dry mass. LMA was calculated as the ratio of dry mass to leaf area, while EWT was computed as the difference between fresh and dry mass, normalized by leaf area. A total of 490 leaf samples were collected for analysis, and the proportion of samples across different crops is shown in **Figure 1b**.

### 2.2 Statistical description of plant traits

**Figure 2:**
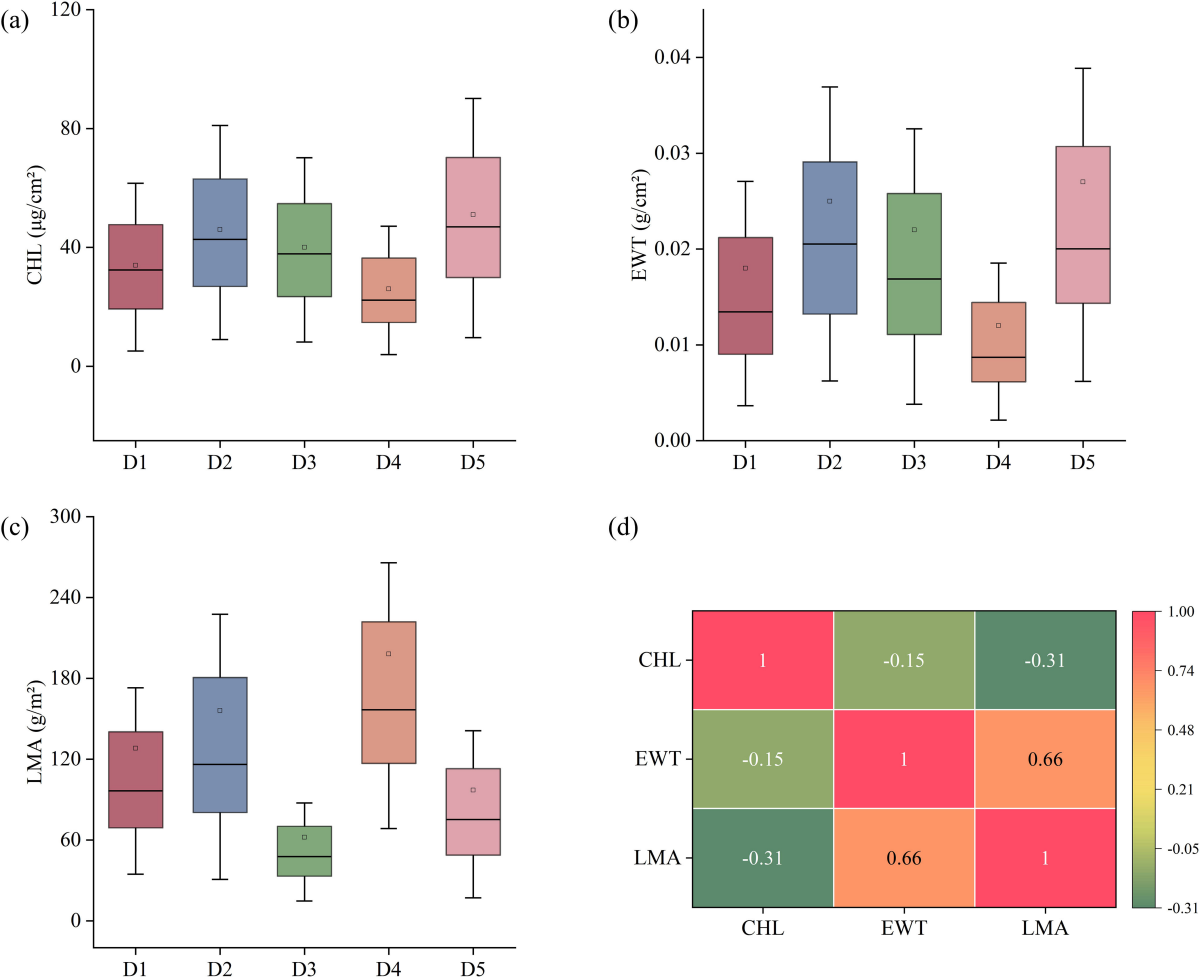
**(a-c)** Distribution of chlorophyll content (CHL), equivalent water thickness (EWT) and leaf mass per area (LMA) for each dataset, **(d)** Pearson correlation coefficient between traits.

The distribution patterns of CHL, EWT, and LMA are illustrated in **Figure 2**. CHL exhibited significant variation among the datasets, with the highest mean value observed in D5 and the lowest in D4. A similar pattern was observed for EWT, where D5 showed the highest values, while D1 and D4 had comparatively lower values. In contrast, LMA exhibited a distinct distribution trend, with D4 showing the highest values and D3 the lowest, indicating substantial variability in LMA across datasets. Additionally, the correlation coefficients among the traits differ: EWT and LMA exhibit a strong positive correlation (0.66), CHL and LMA demonstrate a moderate negative correlation (-0.31), and CHL and EWT show a relatively weak negative correlation (-0.15).

### 2.3 Radiative transfer model

**Figure 3:**
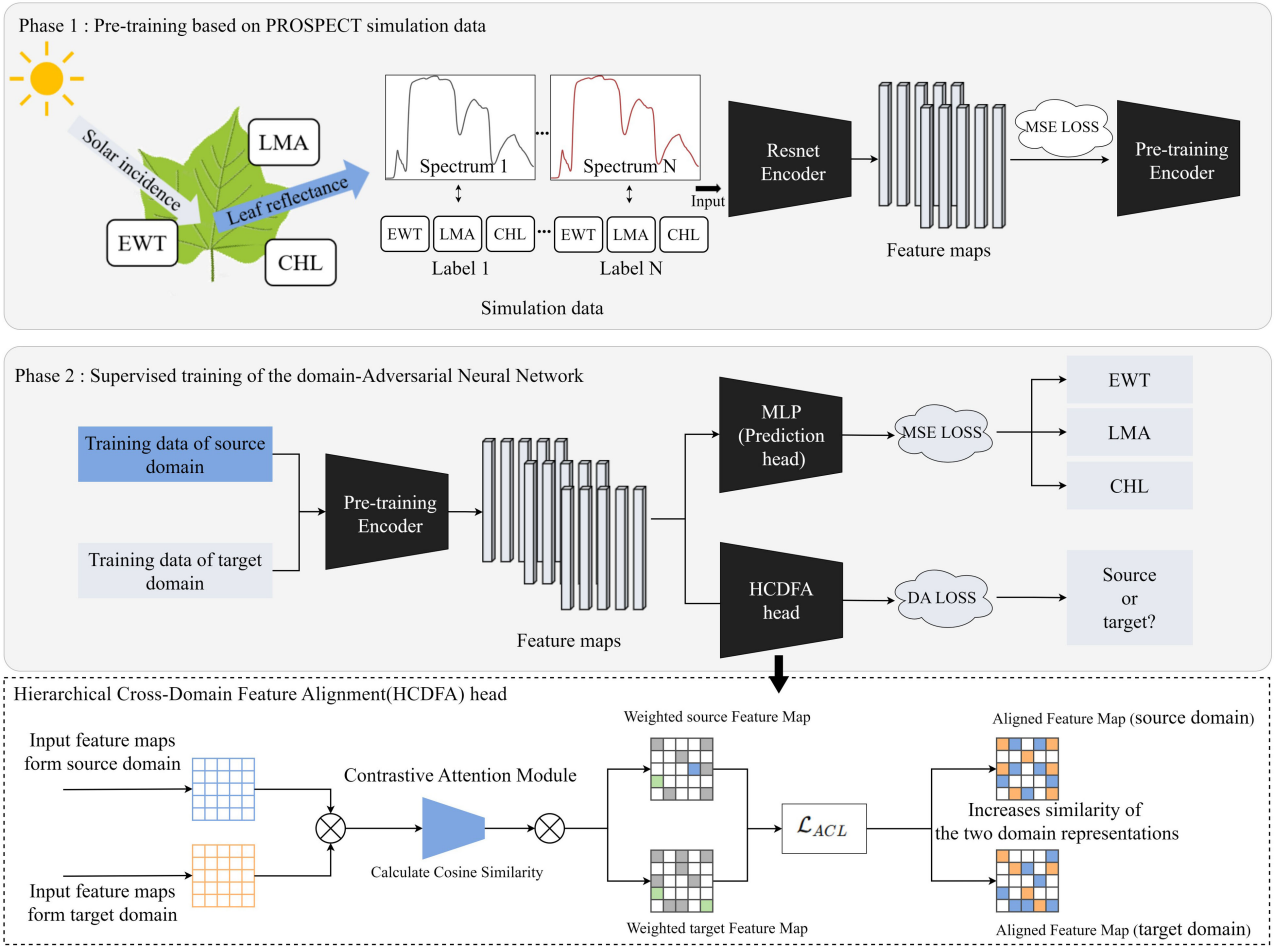
PPADA-Net framework for plant traits prediction. The pre-trained encoder in Phase 1 undergoes supervised fine-tuning in Phase 2. MLP, Multilayer Perceptron; MSE loss, Mean Squared Error loss; DA loss, Domain Adaptation loss; ACL, Adversarial Contrastive Loss.

This study proposes a PROSPECT Pre-training Adversarial Domain Adaptation Network (PPADA-Net), which employs a two-stage training strategy for plant trait prediction (**Figure 3**). The core idea is to fully leverage large-scale simulated spectral data generated by the PROSPECT physical model for pretraining, followed by adaptive fine-tuning across domains using a limited number of target-domain samples.

The PROSPECT radiative transfer model (version PROSPECT-D) has been employed to generate high-fidelity leaf spectral simulation data (Feret et al., 2017). Based on physical optics principles, the PROSPECT model simulates leaf reflectance and transmittance spectra across 400–2400 nm by coupling anatomical structure parameters with biochemical parameters. Through radiative transfer equations in layered media, this model quantitatively characterizes multiple scattering and absorption effects within leaves, effectively capturing the nonlinear influence of various plant traits on spectral responses. A full-spectrum simulation dataset was established using the PROSPECT-D model to generate 20,000 synthetic spectral-trait pairs for pre-training. The model simulates leaf reflectance and transmittance across 400–2400 nm at 1 nm resolution, based on biophysical parameters: CHL (0.1–100 µg/cm²), leaf water depth (0.01–0.05 cm), and dry matter content (0.004–0.009 g/cm²), with additional modulators like total anthocyanin content (1.2-1.8) and leaf structure index (1.0–1.9). Leaf water depth and dry matter content can be converted to EWT and LMA, respectively, enabling direct mapping between physical parameters and functional traits. A Latin hypercube sampling strategy ensured uniform parameter sampling, covering biologically plausible ranges (**Table 2**). Each sample comprises a reflectance spectrum (400–2400 nm) paired with corresponding CHL, EWT, and LMA values, mimicking diverse ecosystem conditions.

**Table 2:** Overview of the PROSPECT-D input variables of plants.

Variable Name	Symbol	Unit	Typical Range
Leaf structure index	*N*	Unitless	1.0-1.9
Chlorophyll a+b content	$C_{ab}/LCC$	$\mu \text{g}/{\text{cm}}^{2}$	0.1-100
Total carotenoid content	$C_{cx}$	$\mu \text{g}/{\text{cm}}^{2}$	1.0-25.0
Total anthocyanin content	$C_{an}$	$\mu \text{g}/{\text{cm}}^{2}$	1.2-1.8
Brown pigments	$C_{\text{bp }}$	Unitless	0.01-1.0
Dry matter content	$C_{m}$	$\text{g}/{\text{cm}}^{2}$	0.004 – 0.009
Leaf water depth	$C_{w}$	cm	0.01 - 0.05

### 2.4 Proposed PPADA-net

**Figure 4:**
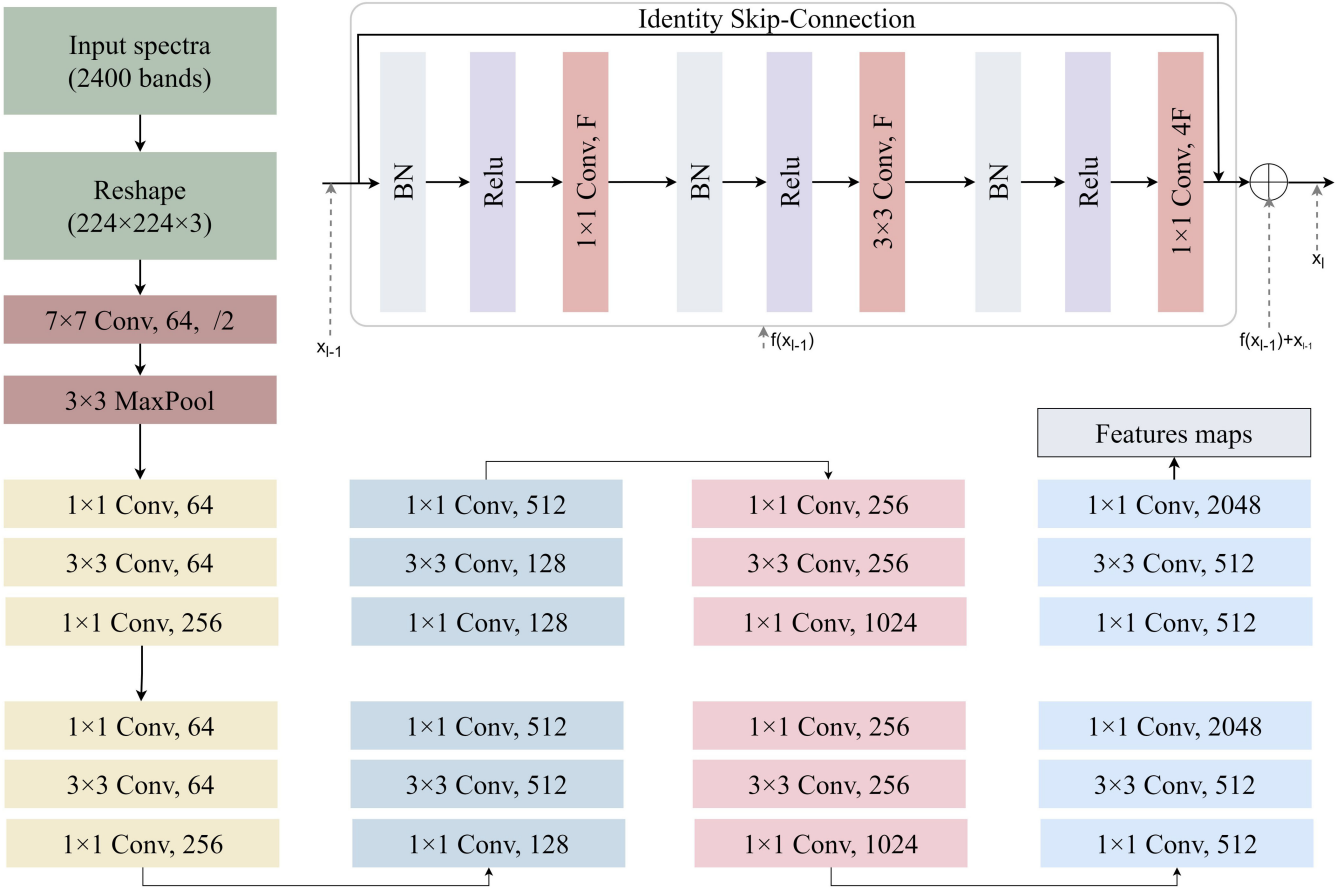
A schematic illustration of 2D ResNet-18 architectures used for yield prediction in this study. Each convolutional layer is followed by batch normalization and a ReLU.


The simulated spectra are fed into a ResNet-based encoder network to extract high-level spectral features (**Figure 4**). The architecture processes spectral inputs through a hierarchical feature extraction pipeline. The hyperspectral reflectance data consisted of 2000 bands (400–2400 nm at 1 nm resolution). To enable compatibility with ResNet, the 1D spectra were reshaped into 2D arrays of size 224×224 and duplicated across three channels to form 224×224×3 tensors. This operation preserves spectral information and allows the use of 2D convolutional layers, which are effective in capturing local and hierarchical patterns. This transformation is purely structural and preserves the original spectral information. The network commences with a 7×7 convolutional layer (64 filters, stride=2) followed by 3×3 max-pooling, establishing preliminary spatial-spectral representations. Subsequent residual blocks employ bottleneck structures with cascaded 1×1 and 3×3 convolutions, progressively expanding channel dimensions from 64 to 1024 through four major stages. Each stage contains multiple identity-short cut blocks where 1×1 convolutions perform channel dimension matching, while 3×3 convolutions extract spatially invariant features. Notably, the architecture implements channel scaling factors of ×4 between stages (64→256→512→1024), maintaining computational efficiency through bottleneck compression. The deep stack of 21 convolutional layers leverages residual connections to preserve gradient flow, with feature map spatial resolution systematically reduced through stride convolutions in transitional blocks. This design enables effective learning of multiscale spectral-spatial correlations while mitigating vanishing gradient issues inherent to deep networks. At this stage, a fully connected regression head is appended to the encoder output. The final encoder outputs high-dimensional latent representations suitable for downstream regression tasks through attached task-specific heads. We optimize the network by minimizing the mean squared error (MSE) loss function (LeCun et al., 2015) to predict LMA, EWT, and CHL values, with gradient backpropagation throughout the network to learn latent representations strongly correlated with plant traits from the large-scale simulated data. The MSE LOSS function is defined in Equation 1.

(1) $$\begin{array}{c} L_{m}=\frac{1}{M}\sum_{i=1}^{M}{\left(y_{i,m}-{\hat{y}}_{i,m}\right)}^{2} \end{array}$$

where m represents the trait index, and M represents the number of samples. The loss for combining all traits is defined in Equation 2.


(2) $$\begin{array}{c} L_{\text{downstream tasks }}=\sum_{m=1}^{T}L_{m} \end{array}$$

Following pre-training, the second stage involves jointly feeding both the source domain (real-world dataset for training) and limited target domain (real-world dataset for prediction) into the pre-trained encoder. This strategy leverages the spectral feature representation capabilities acquired from large-scale simulated data while enabling fine-tuning and domain alignment through limited target domain samples, thereby enhancing prediction accuracy in real-world target environments. To simultaneously address plant trait prediction and domain adaptation, we introduce two parallel task heads at the output of the pre-trained encoder: (1) A Multilayer Perceptron (MLP) for regressing three plant traits (LMA, EWT, and CHL) as the downstream task, optimized using MSE loss. (2) A Hierarchical Cross-Domain Feature Alignment (HCDFA) head, this module takes input feature maps from both the source domain and target domain and processes them through a two-step mechanism. First, a Contrastive Attention Module calculates the cosine similarity between the input feature maps, emphasizing shared patterns while suppressing domain-specific noise. This process generates weighted feature maps for both domains. Subsequently, an adversarial contrastive loss ACL is applied to refine these weighted feature maps, increasing the similarity between the aligned feature representations of the source and target domains. These similarity weights are applied to the source domain feature map, dynamically adjusting its feature distribution to produce the aligned source domain feature map. Simultaneously, the target domain features undergo adaptive mapping to generate the aligned target domain feature map. During forward propagation, the HCDFA acts as an identity transform, while in backward propagation it inverts gradients from the DA loss, thereby encouraging the encoder to learn domain-invariant features through adversarial confusion. The composite objective function during this stage comprises two components is defined in Equation 3.


(3) $$\begin{array}{c} L=\lambda_{1}L_{MSE}+\lambda_{2}L_{DA} \end{array}$$

$L_{MSE}$ represents the MSE loss for plant trait regression, while $L_{DA}$ denotes the domain adaptation loss. λ_1_ and λ_2_ are hyperparameters that control the weighting of the two loss terms, and their optimal balance can be determined through hyperparameter tuning on the validation set.

### 2.5 Training parameters

During network training, stochastic gradient descent is used to update the parameters of both the encoder and the two task heads in mini batches. The Few-shot learning strategy incorporates a small amount of labeled target-domain data into the network alongside source-domain data. A feature alignment mechanism mitigates domain shift, facilitating cross-domain knowledge transfer between datasets. Model training was conducted using a staged optimization strategy. In the first stage, pre-training was conducted using PROSPECT-D-generated spectral-trait pairs to initialize the ResNet-based encoder. The network, structured with a 7×7 convolutional layer (64 filters, stride=2), 3×3 max-pooling, and residual blocks scaling channels from 64 to 1024, was trained for 200 epochs with a batch size of 64. The Adam optimizer was used with an initial learning rate of 0.001 for rapid convergence, decaying by 1×10–5 in later stages to prevent overfitting. The MSE loss function guided optimization, enabling the encoder to learn spectral features strongly correlated with CHL, EWT, and LMA, forming a robust foundation for subsequent domain adaptation. In the second stage of transfer learning, under the integration of source and target domain data, the number of epochs was adjusted to 100 with a batch size of 32, and the initial learning rate was updated to 1×10^-4 to facilitate more refined fine-tuning and domain adaptation.

### 2.6 Comparison of prediction models and verification strategies

Four regression methodologies were systematically compared for CHL, EWT, and LMA prediction: (1) conventional multivariate PLSR, (2) data-driven ResNet, along with two ablated variants, (3) physics-enhanced ResNet-PROSPECT and (4) domain-adaptive ResNet-GRL. These were rigorously benchmarked against our proposed PPADA-Net to evaluate the incremental benefits of integrated physical-adversarial learning.

As a conventional multivariate statistical approach, PLSR establishes linear relationships between hyperspectral reflectance (predictors) and plant traits (response variables) through latent variable decomposition. This model maps the relationship between spectral data and plant traits to a low-dimensional latent space, allowing PLSR to perform effective regression analysis while maintaining low computational cost, representing traditional chemometrics methodology in spectral analysis (Wold et al., 2001).

The standard ResNet18 architecture was adapted for spectral regression, employing residual blocks with 1D convolutions to capture hierarchical spectral features. Without any domain adaptation mechanisms, this deep learning baseline utilized raw spectral inputs (400–2400 nm) to directly predict trait values through fully connected regression layers, demonstrating pure data-driven modeling capability (Chen et al., 2022).

ResNet-PROSPECT integrated physics-informed pretraining by initializing weights through simulated data generated from the PROSPECT-D radiative transfer model. The network first underwent 100-epoch pretraining on 10000 synthetic spectra-trait pairs covering the full parameter space, followed by fine-tuning on experimental datasets, testing the isolated effect of physical prior integration.

The ResNet-GRL architecture implements domain adversarial learning without physical constraints by appending a GRL between the feature extractor and the domain classifier. This dual-objective network simultaneously minimizes trait prediction error and maximizes domain confusion through adversarial training (λ = 0.3), thereby evaluating the independent contribution of domain-invariant feature learning.

### 2.7 Performance evaluation

**Figure 5:**
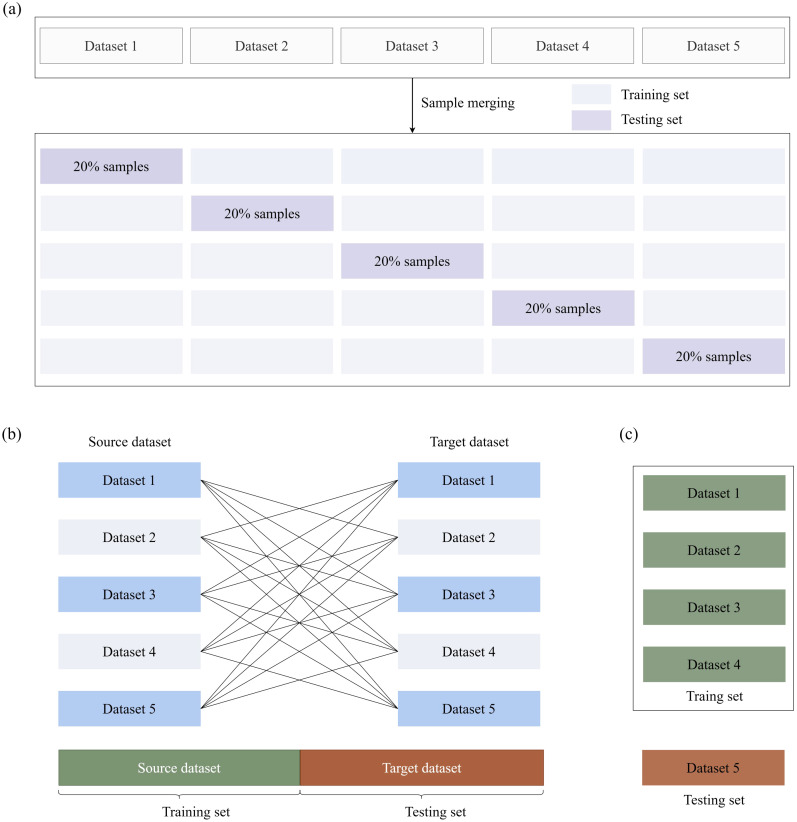
Validation methodologies for plant trait prediction models. **(a)** Combined samples from five datasets (D1–D5) were subjected to stratified five-fold cross-validation. **(b)** Each dataset was used as the validation set in turn, with another single dataset used for training, ensuring robustness across independent datasets. **(c)** D5 was used as the testing set, and the remaining four datasets (D1–D4) were used as the training set.

Two distinct validation paradigms were implemented to comprehensively assess model performance: (1) Aggregated Cross-Validation (**Figure 5a**): All samples from the five datasets (D1-D5) were pooled into a composite repository, followed by a stratified five-fold cross-validation scheme, where each fold maintained proportional representation of the original dataset distributions. In each iteration, 80% of the samples were allocated for training (with 15% reserved for internal validation to facilitate early stopping) and 20% for testing. This approach assessed general predictive accuracy under the assumption of homogeneous data distribution. (2) Cross-Dataset Validation (**Figure 5b**): To rigorously evaluate cross-domain transferability, leave-one-dataset-out experiments were conducted. In each trial, four datasets were used as the training set, while the remaining dataset was held out for independent testing. Spatial-spectral standardization was applied to each dataset using the respective training statistics to prevent information leakage. This protocol specifically quantified the model’s generalization capacity across heterogeneous data acquisition conditions. (3) Training on D1–D4 with Testing on D5 (**Figure 5c**): Datasets D1–D4 were used as the training set, and the model’s predictive performance was tested on the independent, independently collected field dataset D5. This validation methodology is particularly valuable for assessing model robustness and practical applicability in real-world agricultural settings.

In the model evaluation, to measure the variability of the dependent variable and prediction error, the normalized root means square error (nRMSE) and the coefficient of determination (R^2^) are formulated in Equations 4, 5.


(4) $${\text{R}}^{2}=1-\frac{\sum_{i=1}^{n}{\left({\hat{y}}_{i}-y_{i}\right)}^{2}}{\sum_{i-1}^{n}{\left(y_{i}-\bar{y}\right)}^{2}}$$

(5) $$\begin{array}{c} \text{nRMSE }=\frac{1}{n}\sqrt{\frac{1}{\bar{y}}\sum_{i-1}^{n}{\left({\hat{y}}_{i}-y_{i}\right)}^{2}} \end{array}$$

where n is the number of samples; $y_{i}$ and ${\hat{y}}_{i}$ represent the measured and the predicted grain yield of sample i, respectively. A higher value of R^2^ and lower nRMSE values would indicate superior performance of the model.

## 3 Results

### 3.1 Statistical analysis of data

**Figure 6:**
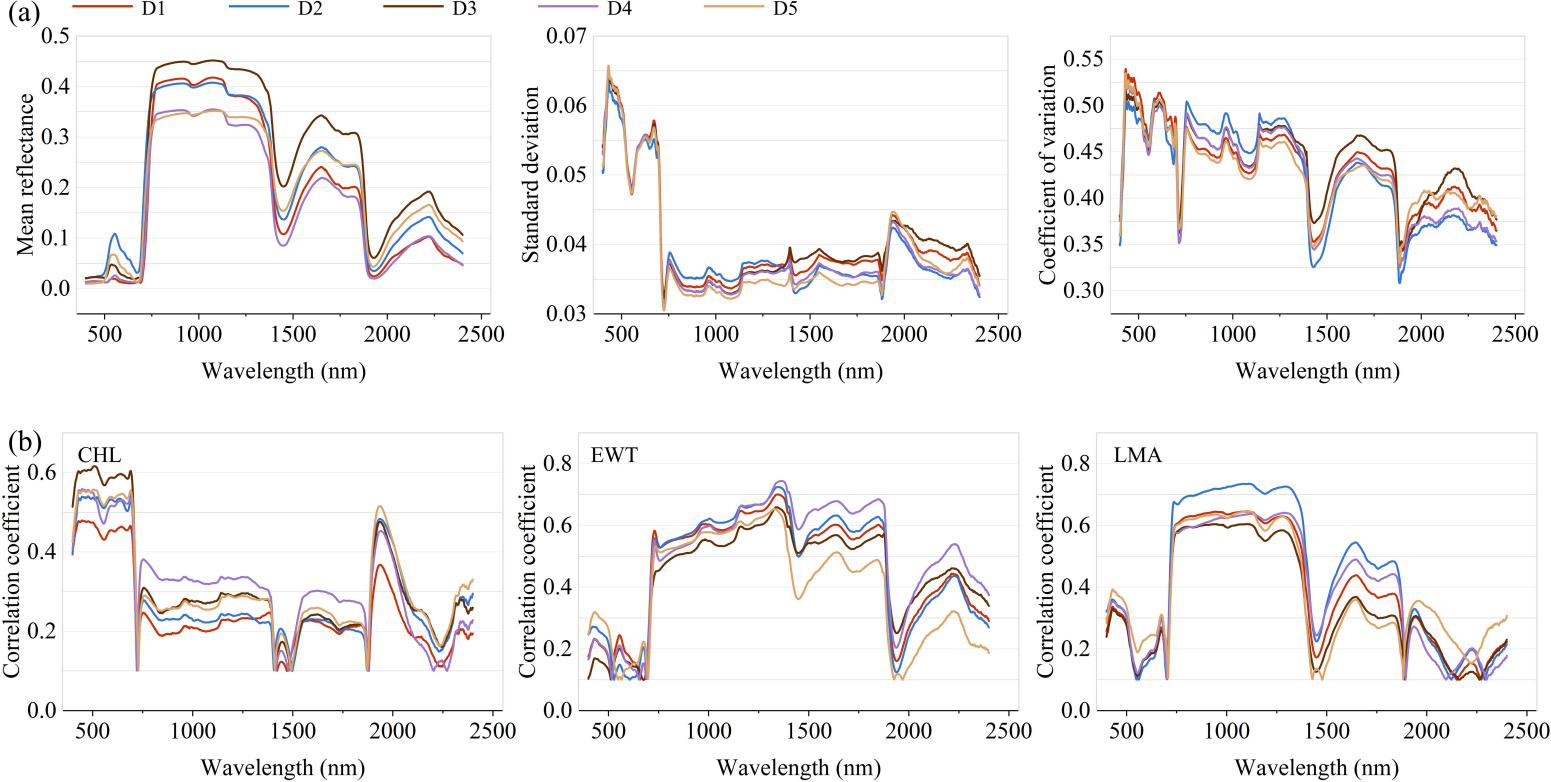
**(a)** Mean, standard deviation and coefficient of variation of spectral reflectance. **(b)** Pearson correlation coefficient between spectral reflectance and traits.

Statistical analyses of spectral reflectance across the 400–2400 nm range are illustrated in **Figure 6a**. Consistent patterns were observed across the five datasets, with all exhibiting identifiable spectral peaks and troughs. In the visible region (500–700 nm), mean reflectance values remained low and relatively stable, reflecting strong pigment absorption. A sharp increase occurred in the near-infrared (NIR, 700–1300 nm), consistent with the high reflectance associated with internal leaf structure. This was followed by a fluctuating decline in the short-wave infrared (SWIR, 1300–2400 nm), where a pronounced dip was observed between 1900 and 2100 nm—likely due to strong water absorption features. Inter-dataset differences were especially evident in specific spectral bands. For instance, the standard deviation of reflectance was notably higher in the visible spectrum, indicating greater variability among datasets in this region. Conversely, variability declined in the NIR and SWIR regions, particularly around 1000–1300 nm and 1900–2100 nm, as shown by the narrower spread in standard deviation and coefficient of variation (CV) curves.

**Figure 6b** presents the Pearson correlation between spectral reflectance and plant traits. For CHL, the strongest correlations were found in the visible spectrum (400–700 nm), aligning with known pigment absorption features. Correlation strength diminished in the NIR and SWIR regions, generally falling below 0.4 except in the 1900–2400 nm sub-region. In contrast, EWT exhibited weak associations in the visible range, but showed moderate to strong correlations (r > 0.5) throughout the 700–1900 nm range, underscoring its sensitivity to water-related absorption features in those bands. For LMA, positive correlations peaked in the 700–1400 nm range, suggesting a strong relationship between biomass-related traits and reflectance in this region. Trait–spectrum relationships also varied across datasets. For example, Dataset D2 exhibited higher LMA correlations in the NIR, while Dataset D5 consistently showed weaker correlations for EWT, reflecting the combined effects of biological variability and environmental conditions. These results emphasize the importance of accounting for both spectral region characteristics and dataset heterogeneity in trait modeling.

### 3.2 Comparison of models

#### 3.2.1 Visualization of learned representation

**Figure 7:**
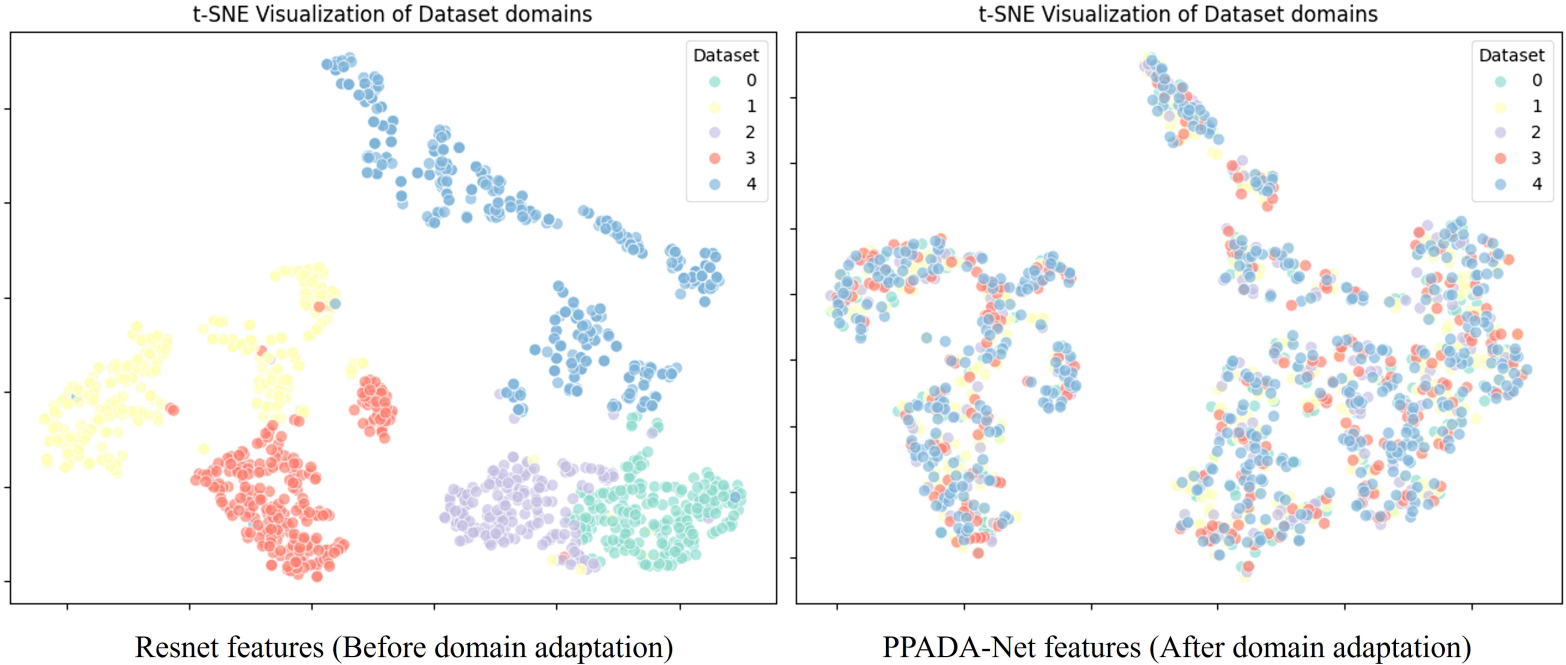
t-SNE visualization of features extracted from different domain datasets.

A t-SNE visualization of feature distributions from multiple datasets is presented in **Figure 7**, comparing features extracted by ResNet with those processed by PPADA-Net using adversarial domain adaptation. The left panel displays ResNet-derived features, where color-coded data points from different datasets form distinct, well-separated clusters with minimal overlap. This sharp separation arises from domain-specific biases in spectral reflectance patterns, leading to distributional mismatches that hinder cross-dataset prediction accuracy in transfer learning. In contrast, the right panel illustrates features processed by PPADA-Net, where adversarial learning induces two key transformations: (1) inter-dataset boundaries become less distinct, with most data points forming mixed-region neighborhoods, and (2) previously compact clusters disperse into overlapping distributions, particularly in high-dimensional manifolds associated with invariant spectral features. These structural adjustments indicate that PPADA-Net effectively mitigates domain shifts by learning transfer-invariant representations, enhancing knowledge transfer across heterogeneous datasets. This alignment mechanism underpins PPADA-Net’s superior cross-domain generalization performance.

#### 3.2.2 Traits prediction performance

**Figure 8:**
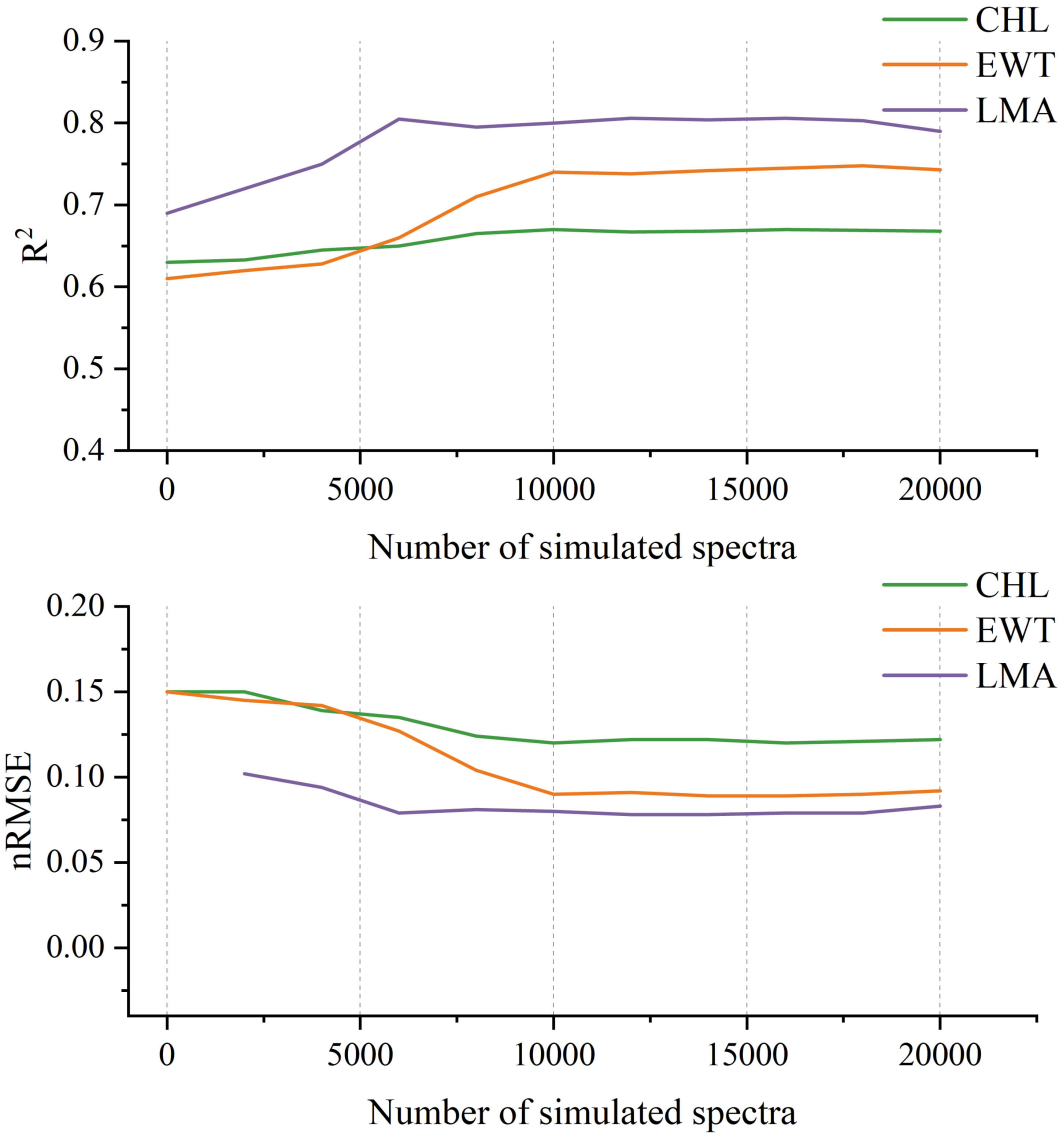
Model performance on three plant traits (CHL, EWT, and LMA) with varying numbers of PROSPECT-simulated spectra used during pretraining.

To evaluate the impact of synthetic data volume, the model was pretrained using varying amounts of PROSPECT-generated spectra (2k, 4k, 6k,…, 20k). As shown in **Figure 8**, predictive accuracy increased with the size of the synthetic dataset. The improvement was particularly notable for CHL and EWT, indicating that PROSPECT provides informative priors related to canopy chlorophyll and water status. Although performance plateaued or showed minor fluctuations beyond 10,000 samples for certain traits, this data volume represents a practical trade-off between model accuracy and computational efficiency.

**Figure 9:**
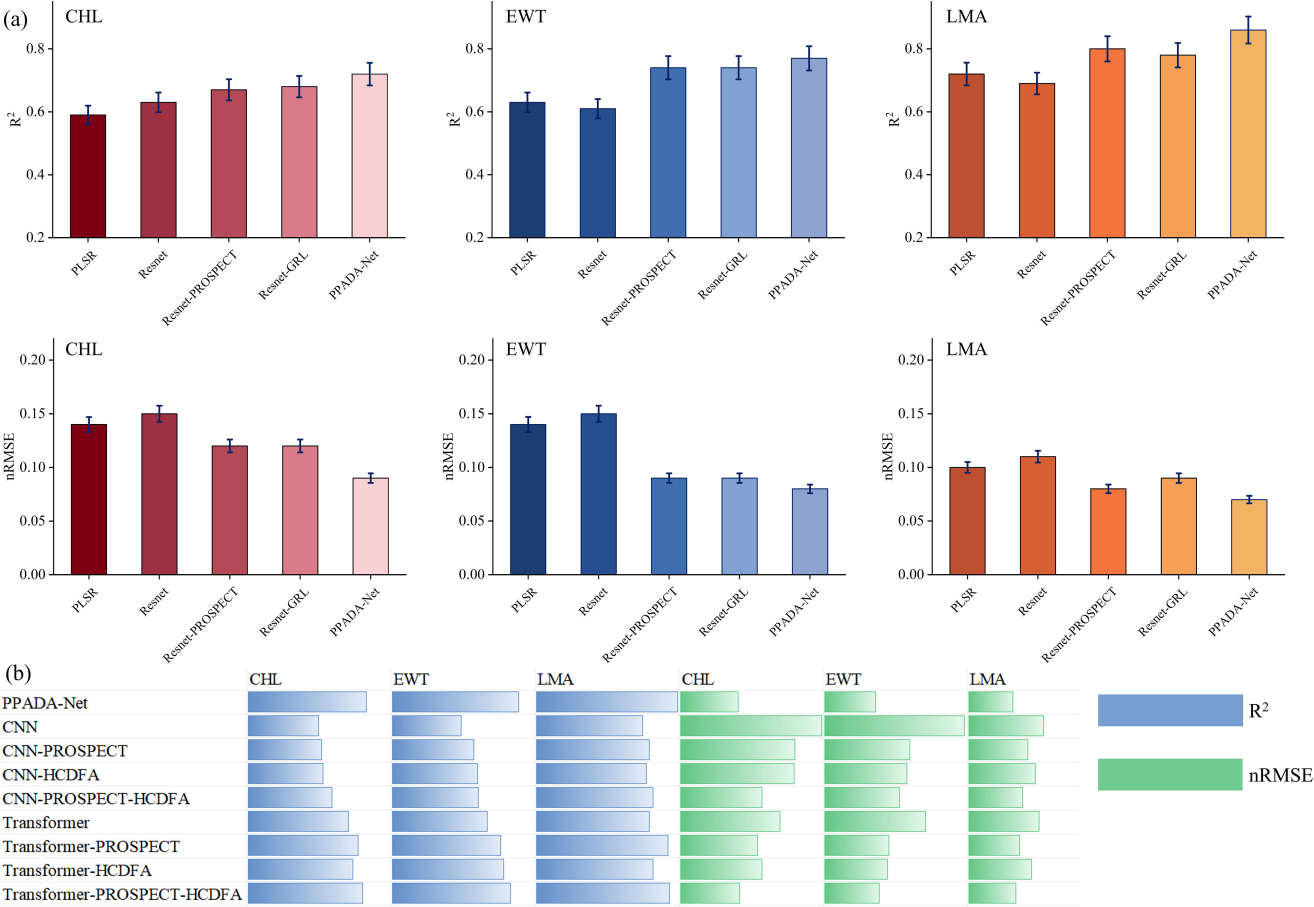
**(a)** Accuracy evaluation of the PLSR, ResNet, ResNet-PROSPECT, ResNet-GRL and PPADA-Net traits prediction models. The error bar represents the standard deviation of the validation. **(b)** uses horizontal bars to compare models like CNN, CNN-PROSPECT, CNN-HCDFA, and Transformers against metrics R² and nRMSE. CHL, Chlorophyll Content; EWT, Equivalent Water Thickness; LMA, Leaf Mass per Area.

This study evaluates the performance of various spectral reflectance prediction models for three plant traits. As illustrated in **Figure 9a**, the models exhibit significant differences in R² and nRMSE. Overall, the traditional machine learning model PLSR demonstrates relatively low accuracy, with average R² values of 0.59, 0.63, and 0.72 for CHL, EWT, and LMA, respectively. In contrast, the deep learning based ResNet improves the R² for CHL to 0.63 (an increase of 6.8%) via nonlinear feature extraction, although its EWT prediction slightly degrades (R² = 0.61 versus 0.63), underscoring the limitations of purely data driven, end to end training for cross trait generalization. Notably, incorporating physical priors and domain adaptation strategies significantly enhances model performance. For example, ResNet-PROSPECT, pretrained on simulated data generated by the PROSPECT radiative transfer model, attains R² values of 0.67, 0.74, and 0.80 for CHL, EWT, and LMA, respectively, which represents an average improvement of 8.3% over the basic ResNet and shows the most substantial error reduction in the EWT prediction (nRMSE = 0.09 versus 0.15). Furthermore, ResNet-GRL employs adversarial learning to align features between the source and target domains, yielding a marginal improvement in CHL prediction (R² = 0.68 versus 0.67) but slightly inferior LMA performance compared to ResNet-PROSPECT (R² = 0.78 versus 0.80), suggesting that pretraining with physically simulated data is more advantageous for certain traits, such as LMA. The dual strategy PPADA-Net, which integrates physical constraints and domain adaptation, achieves the best overall performance by increasing the R² values for CHL, EWT, and LMA to 0.72, 0.77, and 0.86, respectively, which represents an average enhancement of 5.1% over single strategy models and demonstrates balanced improvements across different traits. From the perspective of individual traits, LMA exhibits the highest prediction accuracy (PPADA-Net R² = 0.86), likely due to a more pronounced physical association between its spectral characteristics and dry matter content. The variation in prediction performance across traits aligns with spectral-trait correlations in Section 3.1. LMA exhibits the strongest correlations, especially in the NIR region (700–1400 nm), with stable reflectance and strong biomass associations. EWT displays moderate to strong correlations over a slightly narrower range (700–1900 nm). In contrast, CHL exhibits strong correlations only in the narrow visible range (400–700 nm). These correlation patterns explain why LMA consistently achieves the highest prediction accuracy, followed by EWT, with CHL showing the lowest performance. This underscores the importance of intrinsic spectral sensitivity and the information content available for each trait in determining model accuracy.

The performance of each architecture under three settings: Baseline, PROSPECT pretraining, and with the HCDFA module was analyzed in **Figure 9b**. Overall, ResNet achieved the best prediction accuracy across the three target traits, followed closely by Transformer, while CNN showed a notable performance drop. In ablation analysis, both PROSPECT pretraining and the HCDFA module led to performance improvements across all architectures. When both modules were combined, further accuracy gains were observed, suggesting that PROSPECT provides beneficial physical priors, and the domain alignment strategy of HCDFA effectively mitigates cross-ecosystem trait heterogeneity.

### 3.3 Transferability validation across different datasets

**Figure 10:**
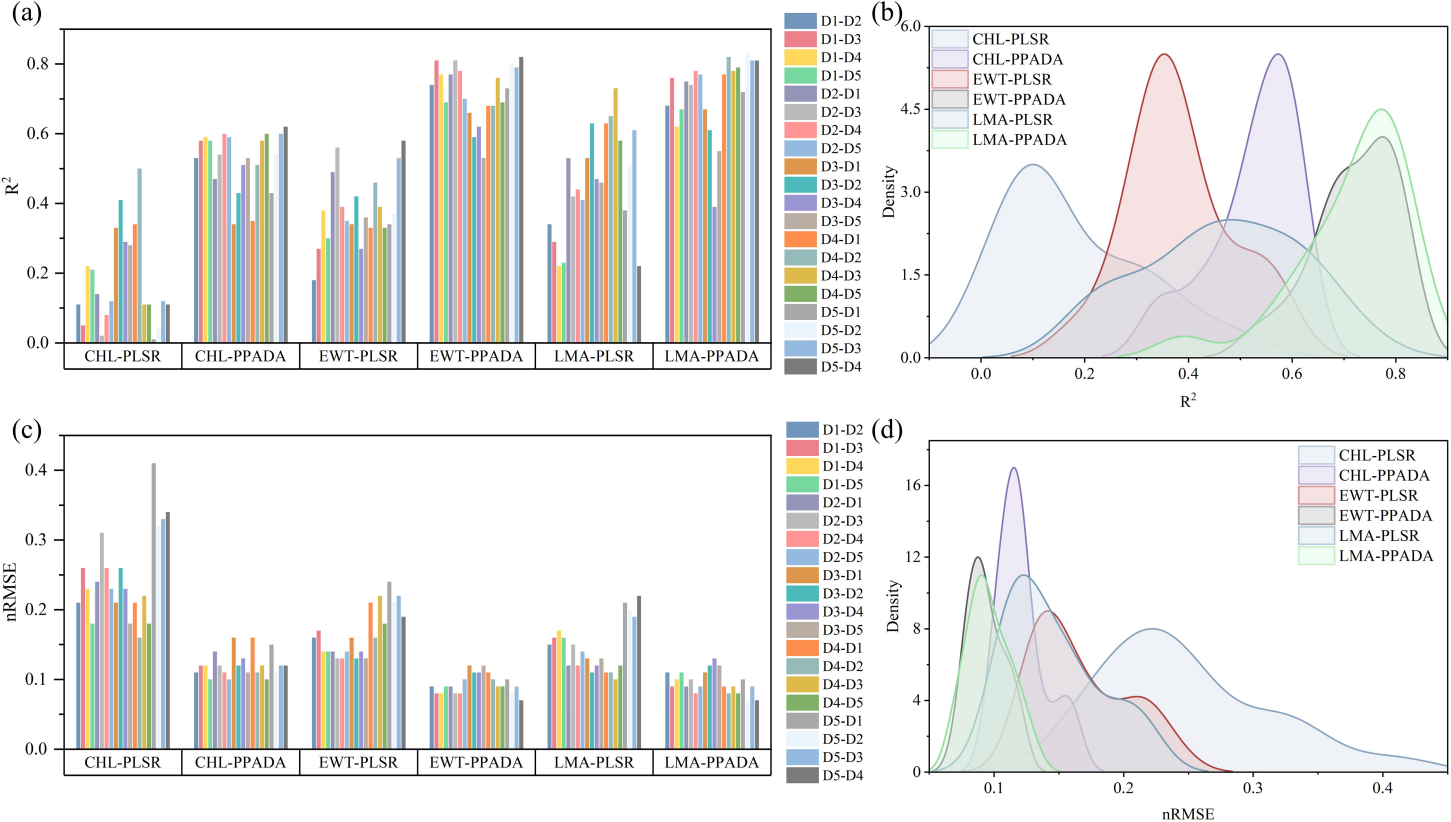
**(a, c)** Cross validation of the PLSR and PPADA-Net traits prediction models (R^2^ and nRMSE). D1–D2 means that Dataset 1 is the training set and Dataset 2 is the test set, **(b, d)** the kernel density estimate (KDE) of the trait-based metric dis tributions (R2 and nRMSE).

The performance of the PLSR model and the proposed PPADA-Net in predicting three plant traits was evaluated (**Figure 10**) across various training-testing dataset combinations (**Tables 3**, **4**). PPADA-Net consistently outperformed the PLSR model across most dataset combinations for all three traits. On average, PPADA-Net exhibited higher R² values and lower nRMSE values than PLSR. For instance, in predicting CHL, PPADA-Net achieved a mean R² of 0.53 and a mean nRMSE of 0.12 across all dataset combinations, whereas PLSR attained a mean R² of 0.18 and a mean nRMSE of 0.25. Similarly, PPADA-Net achieved an average R² of 0.72 and an nRMSE of 0.10 in predicting LMA, whereas PLSR attained an R² of 0.46 and an nRMSE of 0.15. This indicates that PPADA-Net, which integrates simulated data pre-training with adversarial domain alignment, significantly enhances prediction accuracy and reliability.

**Table 3:** The performance results of plant traits prediction at five growth stages using PLSR and PPADA model.

Group	CHL-PLSR	CHL-PPADA	EWT-PLSR	EWT-PPADA	LMA-PLSR	LMA-PPADA
D1-D2	0.11	0.53	0.18	0.74	0.34	0.68
D1-D3	0.05	0.58	0.27	0.81	0.29	0.76
D1-D4	0.22	0.59	0.38	0.77	0.22	0.62
D1-D5	0.21	0.58	0.3	0.69	0.23	0.67
D2-D1	0.14	0.47	0.49	0.77	0.53	0.75
D2-D3	0.02	0.54	0.56	0.81	0.42	0.74
D2-D4	0.08	0.6	0.39	0.78	0.44	0.78
D2-D5	0.12	0.59	0.35	0.7	0.41	0.77
D3-D1	0.33	0.34	0.34	0.66	0.53	0.67
D3-D2	0.41	0.43	0.42	0.59	0.63	0.61
D3-D4	0.29	0.51	0.27	0.62	0.47	0.39
D3-D5	0.28	0.53	0.36	0.53	0.46	0.55
D4-D1	0.34	0.35	0.33	0.68	0.63	0.77
D4-D2	0.5	0.51	0.46	0.68	0.65	0.82
D4-D3	0.11	0.58	0.39	0.76	0.73	0.78
D4-D5	0.11	0.6	0.33	0.69	0.58	0.79
D5-D1	0.01	0.43	0.34	0.73	0.38	0.72
D5-D2	0.04	0.54	0.37	0.8	0.51	0.83
D5-D3	0.12	0.6	0.53	0.79	0.61	0.81
D5-D4	0.11	0.62	0.58	0.82	0.22	0.81
AVG	0.18	0.53	0.38	0.72	0.46	0.72

The metrics reported are R².

**Table 4:** The performance results of plant traits prediction at five growth stages using PLSR and PPADA model.

Group	CHL-PLSR	CHL-PPADA	EWT-PLSR	EWT-PPADA	LMA-PLSR	LMA-PPADA
D1-D2	0.21	0.11	0.16	0.09	0.15	0.11
D1-D3	0.26	0.12	0.17	0.08	0.16	0.09
D1-D4	0.23	0.12	0.14	0.08	0.17	0.1
D1-D5	0.18	0.1	0.14	0.09	0.16	0.11
D2-D1	0.24	0.14	0.14	0.09	0.12	0.09
D2-D3	0.31	0.12	0.13	0.08	0.15	0.1
D2-D4	0.26	0.11	0.13	0.08	0.12	0.08
D2-D5	0.23	0.1	0.14	0.1	0.14	0.09
D3-D1	0.21	0.16	0.16	0.12	0.13	0.11
D3-D2	0.26	0.12	0.13	0.11	0.11	0.12
D3-D4	0.23	0.13	0.14	0.11	0.12	0.13
D3-D5	0.18	0.11	0.13	0.12	0.13	0.12
D4-D1	0.21	0.16	0.21	0.11	0.11	0.09
D4-D2	0.16	0.11	0.16	0.1	0.11	0.08
D4-D3	0.22	0.12	0.22	0.09	0.1	0.09
D4-D5	0.18	0.1	0.18	0.09	0.12	0.08
D5-D1	0.41	0.15	0.24	0.1	0.21	0.1
D5-D2	0.32	0.11	0.21	0.08	0.2	0.07
D5-D3	0.33	0.12	0.22	0.09	0.19	0.09
D5-D4	0.34	0.12	0.19	0.07	0.22	0.07
AVG	0.25	0.12	0.17	0.09	0.15	0.10

The metrics reported are nRMSE.

The model’s performance varied considerably across different dataset combinations. For example, in predicting LMA, when using D1 as the training set and D2 as the testing set, PPADA-Net achieved an R² of 0.68 and an nRMSE of 0.11, whereas PLSR recorded an R² of 0.34 and an nRMSE of 0.15. Similarly, for the D5–D4 combination, PPADA-Net attained an R² of 0.81 and an nRMSE of 0.07, in contrast to PLSR’s R² of 0.22 and an nRMSE of 0.22. These examples further highlight that the prediction accuracy of data-driven models is significantly influenced by the training set. Under these conditions, PPADA-Net demonstrated greater stability and adaptability across various dataset combinations. For example, in predicting EWT across different dataset combinations, PLSR exhibited an R² range of 0.18–0.58 and an nRMSE range of 0.13–0.24. In contrast, PPADA-Net displayed an R² range of 0.53–0.82 and an nRMSE range of 0.07–0.12, indicating that the integration of simulated data pre-training and adversarial domain alignment enables the model to better handle domain shifts and maintain consistent performance across diverse data sources, which is particularly valuable in cases of high data heterogeneity. Overall, among the three traits, CHL is generally more challenging to predict accurately compared to EWT and LMA. Although PPADA-Net achieved relatively high R² values for LMA in certain cases, some dataset combinations still yielded low R² values, suggesting that achieving high-precision transfer for CHL prediction across different datasets remains challenging.

### 3.4 Performance evaluation in independently collected field dataset

**Figure 11:**
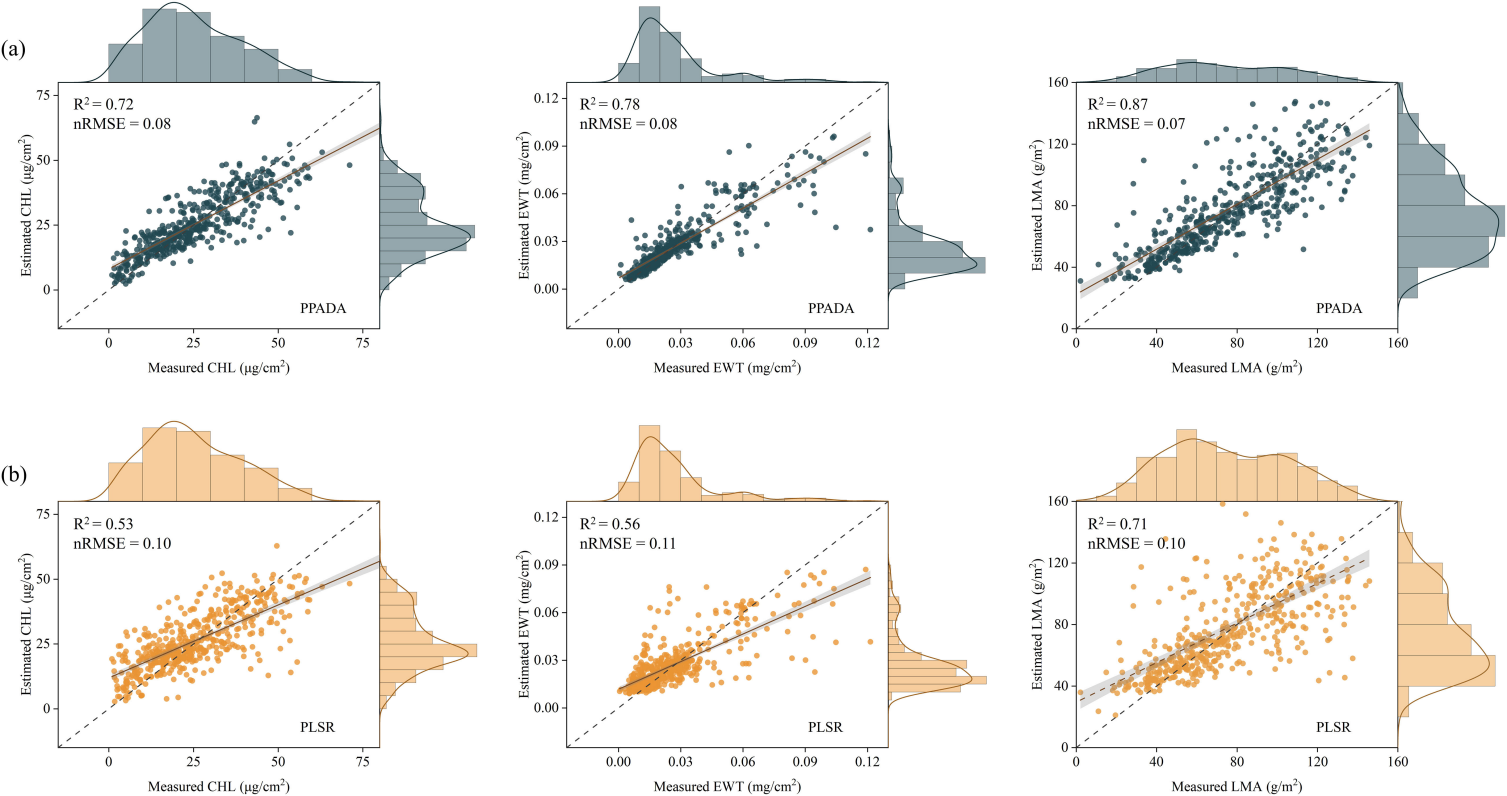
Comparison of prediction performance between the PPADA model **(a)** and PLSR model **(b)** for CHL, EWT and LMA. Each subplot shows scatterplots of estimated versus measured values, with marginal density distributions. The dashed gray line represents the 1:1 line of perfect agreement, and the solid gray line indicates the linear regression fit. The coefficient of determination (R²) and normalized root mean square error (nRMSE) are provided for each model.

When training the model on D1–D4 and testing on the independently collected field D5 dataset, notable performance differences were observed across models. The PPADA model (**Figure 11a**) achieved high prediction accuracy for all three traits, with R² values of 0.72 (CHL), 0.78 (EWT), and 0.87 (LMA), and corresponding nRMSE values of 0.08, 0.08, and 0.07. The scatter plots show strong linear agreement between predicted and observed values, especially for LMA, where most points closely align with the 1:1 line, indicating superior predictive performance. CHL and EWT predictions were more concentrated in the low-to-mid value ranges, with slightly reduced agreement at higher values. In contrast, the PLSR model (**Figure 11b**) produced lower overall accuracy, with R² values of 0.53, 0.56, and 0.71 for CHL, EWT, and LMA, and nRMSE values of 0.10, 0.11, and 0.10, respectively. The scatter plots display greater dispersion, particularly for CHL and EWT, where deviations were more pronounced in the lower measurement ranges. Some improvement in agreement was observed at higher values. Among the three traits, LMA still yielded the best performance, but compared to PPADA, its R² was 0.16 lower and nRMSE was 0.03 higher.

### 3.5 Spatial mapping of plant traits

Accurate mapping of plant functional traits serves as a pivotal tool for advancing crop breeding and precision agricultural management. The PPADA model was trained using datasets D1-D4 and subsequently applied to spatially visualize CHL, EWT, and LMA in 490 field samples from the independent test set D5, as shown in **Figure 11**. The results demonstrate a high degree of consistency between the spatial maps predicted by PPADA and the ground truth measurements, validating the robustness of the model in trait estimation. Among the three traits, LMA exhibited the highest spatial consistency, with prediction errors concentrated in the low-value range. For CHL and EWT, although most predictions closely matched the observed values, the model showed slight underestimation for samples with exceptionally high values. Overall, the PPADA model demonstrated its capability for high-throughput, spectral-based trait monitoring and field mapping applications.

## 4 Discussion

### 4.1 Comparison and analysis of models for plant traits estimation

The use of spectral features, including reflectance data, vegetation indices, and spectral derivatives, for plant trait prediction has been widely applied in previous studies (Li et al., 2024). For example, Aguirre-Gutierrez et al. (2021) demonstrated that key plant functional traits can be accurately predicted across the tropics using the high spatial and spectral resolution of Sentinel-2 imagery in conjunction with climatic and soil information. While PLSR offers high interpretability and computational efficiency in plant traits estimation, it has several limitations. First, it does not support multi-task learning, requiring separate models for different traits, which results in fragmented workflows. Second, it struggles to capture complex spectral-trait relationships, especially in high-dimensional or nonlinear contexts, such as SWIR interactions with leaf dry matter. As shown in our results, PLSR exhibited the lowest performance (mean R² = 0.59–0.72) and showed a significant decline in accuracy in cross-dataset scenarios (e.g., CHL R² = 0.18), highlighting its vulnerability to data heterogeneity.

Deep neural networks, such as CNN, have improved traits prediction by enabling end-to-end multi-task learning and hierarchical feature extraction. Cherif et al. (2023) demonstrated that CNN-based architectures could simultaneously predict multiple traits with enhanced nonlinear modeling. While these models outperform traditional methods in single-domain settings, their heavy reliance on large, labeled datasets poses a challenge (Chen et al., 2023), as training robust models requires extensive field measurements, which are costly and scarce for certain traits. Furthermore, these models often exhibit poor cross-dataset generalizability due to domain shifts in spectral patterns (Zhang and Bao, 2022). Our results support these findings: ResNet showed inconsistent performance across traits (e.g., EWT R² = 0.61 vs. PLSR’s 0.63) and experienced significant accuracy declines in transfer learning scenarios (e.g., LMA R² = 0.68 for D1-D2), highlighting its sensitivity to domain-specific biases. To overcome these limitations, PPADA-Net integrates physical priors from radiative transfer models (e.g., PROSPECT-simulated spectra) with adversarial domain adaptation, achieving dual benefits: physics-informed feature learning and robust cross-domain generalization. It outperformed all baseline models across traits (CHL: R² = 0.72; EWT: R² = 0.77; LMA: R² = 0.86), with accuracy gains of 5.1% to 22.4% over PLSR and ResNet. These results have practical implications for remote sensing-based trait monitoring in real-world settings. PPADA-Net’s strong performance in cross-dataset validation (e.g., LMA R² = 0.72 vs. PLSR’s 0.46) demonstrates its ability to mitigate domain shifts—a key challenge when applying models across regions, time periods, or sensors. This is particularly valuable for large-scale agricultural monitoring and ecosystem management, where collecting labeled data in every new condition is impractical. Furthermore, the integration of physical priors reduces reliance on field data, potentially lowering the cost and labor of trait estimation.

### 4.2 Model performance with PROSEPECT-D simulation data

Physical models, such as the PROSPECT-D radiative transfer model, simulate spectral reflectance based on the biochemical and structural properties of plant leaves, providing mechanistic insights into light-matter interactions (Peters and Noble, 2020). For example, Bhadra et al. (2024) used PROSPECT to simulate hyperspectral responses under varying parameters, demonstrating its effectiveness in controlled experimental settings. While these models offer a rigorous framework for understanding spectral-trait relationships, their standalone application is constrained by computational complexity, sensitivity to input parameter accuracy, and limited adaptability to real-world environmental variability. In contrast, purely data-driven models excel at capturing complex patterns from large datasets but often lack interpretability and struggle with generalization when data is scarce or subject to domain shifts (Hou et al., 2024). Integrating physical models with data-driven approaches has emerged as a promising strategy for improving plant trait estimation.

Integrating PROSPECT-D-generated simulations with empirical datasets mitigates these limitations by leveraging the complementary strengths of both approaches. Physical models enhance training data diversity by synthesizing spectra across a broad range of trait values and environmental conditions, reducing reliance on costly field measurements. Meanwhile, data-driven methods refine feature representations and optimize nonlinear mappings, compensating for the simplifications inherent in physical models. In this study, pretraining ResNet on PROSPECT-D-simulated spectra (ResNet-PROSPECT) significantly improved prediction accuracy compared to the baseline ResNet. For instance, LMA prediction R² increased from 0.72 (ResNet) to 0.80 (ResNet-PROSPECT), while EWT accuracy improved by 17.7% (R²=0.74 vs. 0.63), highlighting the benefits of physics-informed initialization. These improvements result from two key mechanisms: (1) simulated data introduced reflectance variations under extreme or rare trait values, enhancing model robustness; (2) PROSPECT-D’s parameterization guided the network to focus on wavelengths critical for specific traits. The success of PROSPECT-D-enhanced models suggests a scalable approach for trait estimation in heterogeneous environments. By generating synthetic spectra for underrepresented conditions, physical models can pre-train networks to generalize beyond empirical dataset limitations. Future work could explore dynamically integrating physical simulations with domain adaptation, such as iteratively updating PROSPECT-D parameters based on field observations to refine synthetic data quality. Such advancements would further bridge the gap between theoretical modeling and empirical applications, fostering robust solutions for ecosystem monitoring under climate change.

### 4.3 Transferability analysis

The transferability of spectral-trait prediction models is crucial for real-world applications, as ecosystems exhibit substantial variability in spectral signatures and plant trait distributions due to differences in species composition, environmental conditions, and measurement protocols (Zhang et al., 2025a). Traditional machine learning and deep learning models often struggle to generalize across such heterogeneous datasets, limiting their effectiveness in large-scale monitoring. For instance, PLSR, while computationally efficient, demonstrates poor adaptability to spectral heterogeneity. In cross-dataset validation, PLSR achieved a mean R² of only 0.18 for CHL and 0.46 for LMA, with nRMSE values reaching 0.25. Similarly, deep learning models such as ResNet, despite their capacity for multi-task learning, remain susceptible to domain-specific biases.

Adversarial domain adaptation has emerged as a powerful strategy for aligning feature distributions across domains. For example, Ma et al. (2022) proposed a domain adaptation scheme named adversarial entropy optimization to learn domain-invariant features, achieving state-of-the-art performance across diverse domain adaptation tasks. In this study, PPADA-Net integrates this technique with physical priors to address spectral heterogeneity. The model achieved a mean R² of 0.53 for CHL, 0.72 for LMA, and 0.65 for EWT across all dataset combinations, outperforming PLSR by 35–52% in R². Notably, PPADA-Net maintained stable performance even in challenging scenarios, such as the D5→D4 transfer for LMA (R²=0.81 vs. PLSR’s 0.22), demonstrating its robustness against domain-specific noise. The enhanced transferability arises from two synergistic mechanisms. First, PROSPECT-D-simulated spectra provides trait-specific spectral patterns, enabling the model to prioritize domain-invariant biochemical signals over dataset-specific artifacts. Second, by minimizing discrepancies between source and target domains in high-dimensional feature space, PPADA-Net mitigates overfitting to local spectral variations. Future research could extend this framework to dynamically adapt to emerging ecosystems or sensor types, further bridging the gap between controlled simulations and field applications.

### 4.4 Performance on independently collected field datasets

**Figure 12:**
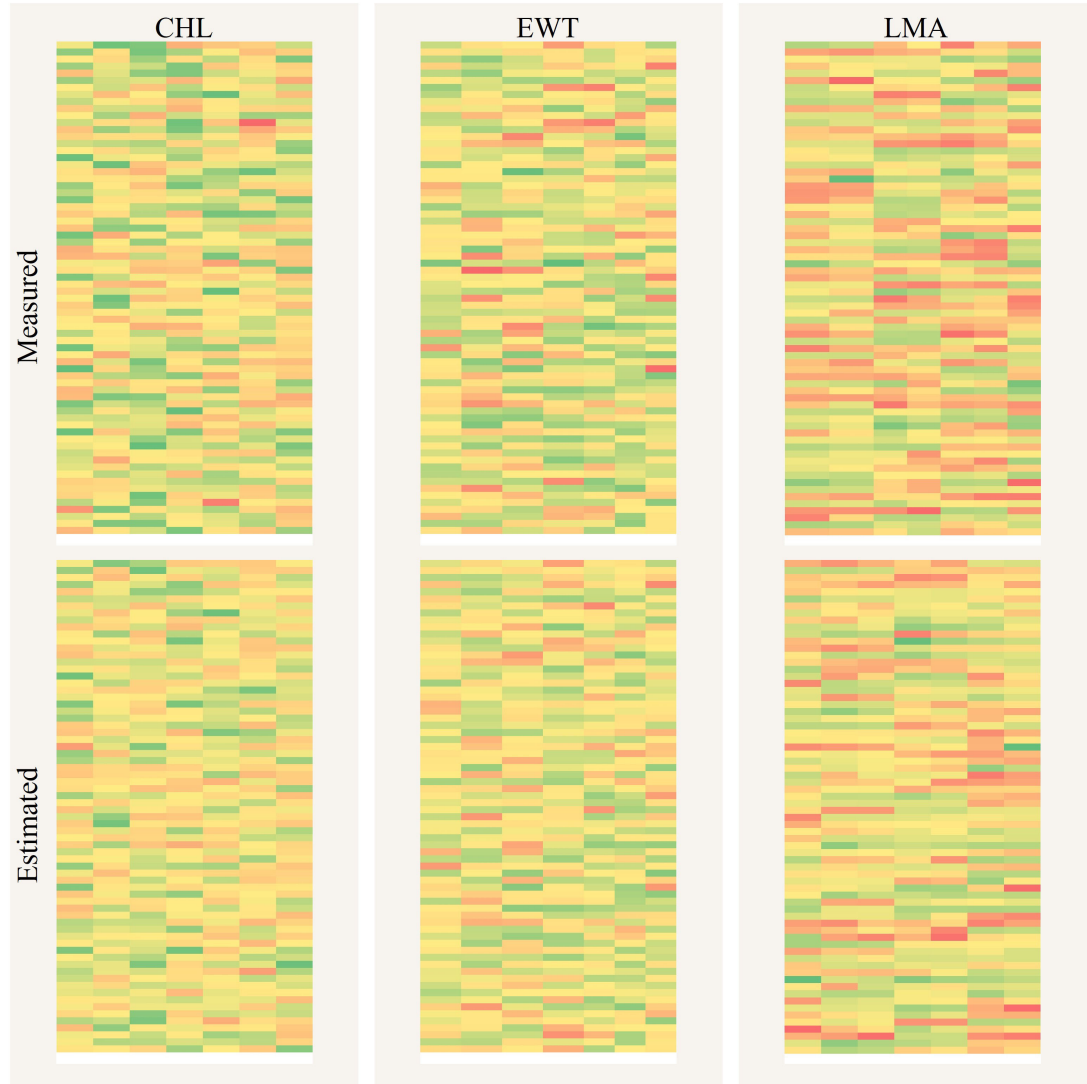
Spatial mapping comparison of plant traits between PPADA predictions and ground measurements. The three above are the measured CHL, EWT, and LMA, while the three below are the predicted values.

PPADA-Net demonstrated exceptional performance on the independently collected field dataset (D5), comprising potato, soybean, and maize crops under diverse cultivars and growth stages. The model achieved R² values of 0.72 (CHL), 0.78 (EWT), and 0.87 (LMA) with nRMSE reductions of 20%–30% compared to PLSR (**Figure 10**). Notably, LMA prediction exhibited the highest accuracy (R² = 0.87), likely due to its strong spectral-physical linkage with dry matter content, as captured by PROSPECT-D simulations. These results underscore the framework’s ability to generalize beyond controlled experimental conditions, addressing the critical challenge of domain shifts caused by cultivar diversity, growth stage variability, and field-specific environmental factors. From an agronomic perspective, the high accuracy of PPADA-Net in mapping LMA and EWT (**Figure 12**) holds significant promise for optimizing irrigation scheduling and nutrient management. For example, spatially resolved LMA estimates could guide breeders in selecting drought-tolerant cultivars, while EWT monitoring may improve water-use efficiency in water-scarce regions. However, slight underestimation of CHL in high-value ranges (**Figure 10a**) suggests that chlorophyll’s nonlinear spectral interactions under saturating conditions require further refinement. Future work should also validate the framework across broader agro-climatic zones and crop phenological stages to ensure scalability.

### 4.5 Limitations

While PPADA-Net demonstrates significant advancements in plant trait estimation, its performance, like other machine learning methods, remains heavily dependent on the quality and quantity of training data. The framework’s effectiveness diminishes when training data are scarce or unrepresentative of target environments. For instance, PPADA-Net’s reliance on PROSPECT-D-generated simulations introduces biases if the radiative transfer model fails to capture extreme environmental conditions. In such cases, synthetic data may inadequately represent real-world spectral-trait relationships, limiting generalization. To mitigate this, future work should incorporate anthropogenic constraints into simulations, such as expanding parameter ranges for leaf structure and biochemical composition, to enhance the diversity and representativeness of synthetic datasets. This would ensure simulated spectra encompass greater information entropy, better aligning with the variability observed in field data. On the other hand, although adversarial learning in PPADA-Net effectively aligns feature distributions across datasets, its performance may degrade when domain discrepancies are extreme. While our results demonstrated robustness in most cross-dataset validations, scenarios involving stark domain shifts require careful hyperparameter tuning to stabilize adversarial training. In summary, by refining the constructive interaction between physically based simulation and adaptive learning, PPADA-Net can evolve into a reliable tool for precision agriculture.

## 5 Conclusion

This study presents the PROSPECT Pre-trained Adversarial Domain Adaptation Network (PPADA-Net), a novel framework that synergizes physical radiative transfer modeling with adversarial domain adaptation to address cross-ecosystem plant trait estimation. Rigorously validated across five datasets—including field experiments conducted at an agricultural research station in Xinxiang, China (D5)—the framework demonstrates robust performance in both controlled and real-world agricultural environments. PPADA-Net achieves state-of-the-art accuracy in predicting chlorophyll content (CHL: R² = 0.72), equivalent water thickness (EWT: R² = 0.78), and leaf mass per area (LMA: R² = 0.87) on the independently measured crop dataset (D5), significantly outperforming conventional PLSR with nRMSE reductions of 20%–30%. Notably, LMA prediction exhibited the highest robustness (nRMSE = 0.07), highlighting the model’s ability to generalize across heterogeneous field conditions, such as cultivar diversity, growth stage variability, and sensor-specific spectral biases. The integration of field-collected hyperspectral data with adversarial domain adaptation proved critical for bridging synthetic simulations and practical applications. For instance, the hierarchical cross-domain alignment module effectively mitigated domain shifts between laboratory datasets (D1–D4) and real-world agricultural data (D5), as evidenced by t-SNE visualizations of domain-invariant features. In summary, PPADA-Net harmonizes physics-driven priors with data-driven adaptability, offering a transformative solution for plant trait estimation in heterogeneous environments.

## Data Availability

The raw data supporting the conclusions of this article will be made available by the authors, without undue reservation.

## References

[B1] Aguirre-Gutierrez J. Rifal S. Shenkin A. Oliveras I. Bentley L. P. Svatek M.. (2021). Pantropical modelling of canopy functional traits using Sentinel-2 remote sensing data. Remote Sens. Environ. 252, 112122. doi: 10.1016/j.rse.2020.112122

[B2] Amirkolaee H. A. Shi M. He L. Mulligan M. (2024). AdaTreeFormer: Few shot domain adaptation for tree counting from a single high-resolution image. Isprs J. Photogramm. Remote Sens. 214, 193–208. doi: 10.1016/j.isprsjprs.2024.06.015

[B3] Angel Y. Shiklomanov A. N. (2022). Remote detection and monitoring of plant traits: theory and practice. Annu. Plant Rev. Online 5.

[B4] Bhadra S. Sagan V. Sarkar S. Braud M. Mockler T. C. Eveland A. L. (2024). PROSAIL-Net: A transfer learning-based dual stream neural network to estimate leaf chlorophyll and leaf angle of crops from UAV hyperspectral images. Isprs J. Photogramm. Remote Sens. 210, 1–24. doi: 10.1016/j.isprsjprs.2024.02.020

[B5] Broge N. H. Leblanc E. (2001). Comparing prediction power and stability of broadband and hyperspectral vegetation indices for estimation of green leaf area index and canopy chlorophyll density. Remote Sens. Environ. 76, 156–172. doi: 10.1016/S0034-4257(00)00197-8

[B6] Chen H. Qi Z. Shi Z. (2022). Remote sensing image change detection with transformers. IEEE Trans. Geosci. Remote Sens. 60, 1–14. doi: 10.1109/TGRS.2021.3095166

[B7] Chen T. Shen M. Guo L. Hu X. (2023). A gridless DOA estimation algorithm based on unsupervised deep learning. Digital Signal Process. 133, 103823. doi: 10.1016/j.dsp.2022.103823

[B8] Cherif E. Feilhauer H. Berger K. Dao P. D. Ewald M. Hank T. B.. (2023). From spectra to plant functional traits: Transferable multi-trait models from heterogeneous and sparse data. Remote Sens. Environ. 292, 113580. doi: 10.1016/j.rse.2023.113580

[B9] Drenovsky R. E. Grewell B. J. D’Antonio C. M. Funk J. L. James J. J. Molinari N.. (2012). A functional trait perspective on plant invasion. Ann. Bot. 110, 141–153. doi: 10.1093/aob/mcs100, PMID: 22589328 PMC3380596

[B10] Fatichi S. Pappas C. Zscheischler J. Leuzinger S. (2019). Modelling carbon sources and sinks in terrestrial vegetation. New Phytol. 221, 652–668. doi: 10.1111/nph.15451, PMID: 30339280

[B11] Feret J. B. Gitelson A. A. Noble S. D. Jacquemoud S. (2017). PROSPECT-D: Towards modeling leaf optical properties through a complete lifecycle. Remote Sens. Environ. 193, 204–215. doi: 10.1016/j.rse.2017.03.004

[B12] Ganin Y. Ustinova E. Ajakan H. Germain P. Larochelle H. Laviolette F.. (2016). Domain-adversarial training of neural networks. J. Mach. Learn. Res. 17, 1–35.

[B13] Haboudane D. Miller J. R. Pattey E. Zarco-Tejada P. J. Strachan I. B. (2004). Hyperspectral vegetation indices and novel algorithms for predicting green LAI of crop canopies: Modeling and validation in the context of precision agriculture. Remote Sens. Environ. 90, 337–352. doi: 10.1016/j.rse.2003.12.013

[B14] Heidenreich K. M. Richardson T. L. (2020). Photopigment, absorption, and growth responses of marine cryptophytes to varying spectral irradiance. J. Phycol. 56, 507–520. doi: 10.1111/jpy.12962, PMID: 31876286

[B15] Helsen K. Bassi L. Feilhauer H. Kattenborn T. Matsushima H. Van Cleemput E.. (2021). Evaluating different methods for retrieving intraspecific leaf trait variation from hyperspectral leaf reflectance. Ecol. Indic. 130, 108111. doi: 10.1016/j.ecolind.2021.108111

[B16] Hoeppner J. M. Skidmore A. K. Darvishzadeh R. Heurich M. Chang H.-C. Gara T. W. (2020). Mapping canopy chlorophyll content in a temperate forest using airborne hyperspectral data. Remote Sens. 12, 3573. doi: 10.3390/rs12213573

[B17] Hou J. Xu J. Lin C. Jiang D. Mei X. (2024). State of charge estimation for lithium-ion batteries based on battery model and data-driven fusion method. Energy 290, 130056. doi: 10.1016/j.energy.2023.130056

[B18] Hunt E. R. Rock B. N. (1989). Detection of changes in leaf water-content using near-infrared and middle-infrared reflectances. Remote Sens. Environ. 30, 43–54.

[B19] Jacquemoud S. Baret F. (1990). Prospect - A model of leaf optical-properties spectra. Remote Sens. Environ. 34, 75–91. doi: 10.1016/0034-4257(90)90100-Z

[B20] Jing J. J. Liu L. Y. Wang J. H. Wang J. D. Zhao C. J. IEEE (2004). “Uncertainty analysis for NDVI using the physical models,” in IEEE International Geoscience and Remote Sensing Symposium(Anchorage, AK) 6, 4321–4324.

[B21] LeCun Y. Bengio Y. Hinton G. (2015). Deep learning. Nature 521, 436–444. doi: 10.1038/nature14539, PMID: 26017442

[B22] Li W. Zuo X. Liu Z. Nie L. Li H. Wang J.. (2024). Predictions of *Spartina alterniflora* leaf functional traits based on hyperspectral data and machine learning models. Eur. J. Remote Sens. 57, 2294951. doi: 10.1080/22797254.2023.2294951

[B23] Ma A. Li J. Lu K. Zhu L. Shen H. T. (2022). Adversarial entropy optimization for unsupervised domain adaptation. IEEE Trans. Neural Networks Learn. Syst. 33, 6263–6274. doi: 10.1109/TNNLS.2021.3073119, PMID: 33939616

[B24] Meloni D. A. Oliva M. A. Martinez C. A. Cambraia J. (2003). Photosynthesis and activity of superoxide dismutase, peroxidase and glutathione reductase in cotton under salt stress. Environ. Exp. Bot. 49, 69–76. doi: 10.1016/S0098-8472(02)00058-8

[B25] Pan S. J. Yang Q. (2010). A survey on transfer learning. IEEE Trans. Knowledge Data Eng. 22, 1345–1359. doi: 10.1109/TKDE.2009.191

[B26] Peters R. D. Noble S. D. (2020). Sensitivity and correlation analysis of PROSPECT-D and ABM-B leaf models. IEEE Trans. Geosci. Remote Sens. 58, 8258–8267. doi: 10.1109/TGRS.36

[B27] Poorter H. Niinemets U. Poorter L. Wright I. J. Villar R. (2009). Causes and consequences of variation in leaf mass per area (LMA): a meta-analysis. New Phytol. 182, 565–588. doi: 10.1111/j.1469-8137.2009.02830.x, PMID: 19434804

[B28] Radford A. Kim J. W. Hallacy C. Ramesh A. Goh G. Agarwal S.. (2021). “Learning transferable visual models from natural language supervision,” in International Conference on Machine Learning (ICML) (Electr Network). 8748–8763.

[B29] Shu M. Dong Q. Fei S. Yang X. Zhu J. Meng L.. (2022). Improved estimation of canopy water status in maize using UAV-based digital and hyperspectral images. Comput. Electron. Agric. 197, 106982.

[B30] Sun G. Zhang Y. Chen H. Wang L. Li M. Sun X.. (2024). Improving soybean yield prediction by integrating UAV nadir and cross-circling oblique imaging. Eur. J. Agron. 155, 127134. doi: 10.1016/j.eja.2024.127134

[B31] Sun G. Zhang Y. Wang L. Zhou L. Fei S. Han S.. (2025). Bridging the gap between hyperspectral imaging and crop breeding: soybean yield prediction and lodging classification with prototype contrastive learning. Comput. Electron. Agric. 230, 109859. doi: 10.1016/j.compag.2024.109859

[B32] Wang Z. Skidmore A. K. Darvishzadeh R. Heiden U. Heurich M. Wang T. (2015). Leaf nitrogen content indirectly estimated by leaf traits derived from the PROSPECT model. IEEE J. Selected Topics Appl. Earth Observ. Remote Sens. 8, 3172–3182.

[B33] Wold S. Sjöström M. Eriksson L. (2001). PLS-regression:: a basic tool of chemometrics. Chemometrics Intelligent Lab. Syst. 58, 109–130. doi: 10.1016/S0169-7439(01)00155-1

[B34] Wright I. J. Reich P. B. Westoby M. Ackerly D. D. Baruch Z. Bongers F.. (2004). The worldwide leaf economics spectrum. Nature 428, 821–827. doi: 10.1038/nature02403, PMID: 15103368

[B35] Yin H. Huang W. Li F. Yang H. Li Y. Hu Y.. (2023). Multi-temporal UAV imaging-based mapping of chlorophyll content in potato crop. Pfg J. Photogramm. Remote Sens. Geoinform. Sci. 91, 91–106.

[B36] Yue Z. Zhang Q. Zhu X. Zhou K. (2024). Chlorophyll content estimation of *ginkgo* seedlings based on deep learning and hyperspectral imagery. Forests 15, 2010. doi: 10.3390/f15112010

[B37] Zhang B. Bao Y. (2022). Cross-dataset learning for age estimation. IEEE Access 10, 24048–24055. doi: 10.1109/ACCESS.2022.3154403

[B38] Zhang R. Cao Z. Huang Y. Yang S. Xu L. Xu M. (2025a). Visible-infrared person re-identification with real-world label noise. IEEE Trans. Circuits Syst. Video Technol. doi: 10.1109/TCSVT.2025.3526449

[B39] Zhang R. Xu L. Yu Z. Shi Y. Mu C. Xu M. (2021). Deep-IRTarget: An automatic target detector in infrared imagery using dual-domain feature extraction and allocation. IEEE Trans. Multimedia 24, 1735–1749. doi: 10.1109/TMM.2021.3070138

[B40] Zhang R. Yang B. Xu L. Huang Y. Xu X. Zhang Q.. (2025b). A benchmark and frequency compression method for infrared few-shot object detection. IEEE Trans. Geosci. Remote Sens. doi: 10.1109/TGRS.2025.3540945

