# Supplementary material for: Integrating PROSPECT-D physics and adversarial domain adaptation resnet for robust cross-ecosystem plant traits estimation
Source: Front Plant Sci. 2025 Jul 25;16:1612430. doi: 10.3389/fpls.2025.1612430 (PMC12331702; doi:10.3389/fpls.2025.1612430)
Supplement: Supplementary file 1 [file Table1.docx]

Appendix table 1 Nomenclature

| Symbol/Abbreviation | Description | Unit (if applicable) |
| --- | --- | --- |
| CHL | Chlorophyll Content | - |
| EWT | Equivalent Water Thickness | g/cm² |
| LMA | Leaf Mass per Area | $g/m^{2}$ |
| PPADA-Net | PROSPECT-Pretrained Adversarial Domain Adaptation Network | - |
| PROSPECT-D | Radiative Transfer Model for Leaf Optical Properties | - |
| ResNet | Residual Network | - |
| PLSR | Partial Least Squares Regression | - |
| HCDFA | Hierarchical Cross-Domain Feature Alignment | - |
| ACL | Adversarial Contrastive Loss | - |
| MSE | Mean Squared Error | - |
| nRMSE | Normalized Root Mean Square Error | - |
| D1-D5 | Datasets 1 to 5, representing various ecosystems | - |
| NIR | Near-Infrared Region (700-1300 nm) | nm |
| SWIR | Short-Wave Infrared Region (1300-2400 nm) | nm |
